# Improved CaP Nanoparticles for Nucleic Acid and Protein Delivery to Neural Primary Cultures and Stem Cells

**DOI:** 10.1021/acsnano.3c09608

**Published:** 2024-01-29

**Authors:** Yu-Wen Chao, Yen-Lurk Lee, Ching-San Tseng, Lily Ueh-Hsi Wang, Kuo-Chiang Hsia, Huatao Chen, Jean-Michel Fustin, Sayma Azeem, Tzu-Tung Chang, Chiung-Ya Chen, Fan-Che Kung, Yi-Ping Hsueh, Yi-Shuian Huang, Hsu-Wen Chao

**Affiliations:** † Department of Physiology, School of Medicine, College of Medicine, Taipei Medical University, Taipei 110301, Taiwan; ‡ Graduate Institute of Medical Sciences, College of Medicine, Taipei Medical University, Taipei 110301, Taiwan; § Department of Anatomy, School of Medicine, China Medical University, Taichung 40402, Taiwan; ∥ Institute of Molecular Biology, Academia Sinica, Taipei 115201, Taiwan; ⊥ Institute of Biomedical Sciences, Academia Sinica, Taipei 115201, Taiwan; # Department of Clinical Veterinary Medicine, College of Veterinary Medicine, Northwest A&F University, Yangling, Shaanxi 712100, China; ¶ Key Laboratory of Animal Biotechnology of the Ministry of Agriculture and Rural Affairs, Northwest A&F University, Yangling, Shaanxi 712100, China; ■ The University of Manchester, Faculty of Biology, Medicine and Health, Oxford Road, Manchester M13 9PL, U.K.; ● Taiwan International Graduate Program in Interdisciplinary Neuroscience, National Yang-Ming Chao-Tung University and Academia Sinica, Taipei 115201, Taiwan; ▲ Institute of Molecular Medicine, College of Medicine, National Taiwan University, Taipei 10002, Taiwan; ▼ Department of Biomedical Science and Environmental Biology, Kaohsiung Medical University, Kaohsiung 80708, Taiwan

**Keywords:** calcium phosphate, nanoparticles, transfection, gene delivery, primary neurons, neural stem cells

## Abstract

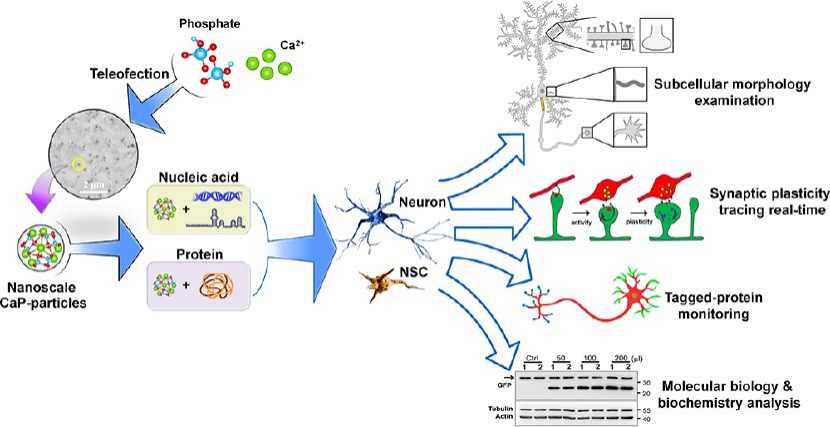

Efficiently delivering exogenous materials into primary neurons and neural stem cells (NSCs) has long been a challenge in neurobiology. Existing methods have struggled with complex protocols, unreliable reproducibility, high immunogenicity, and cytotoxicity, causing a huge conundrum and hindering in-depth analyses. Here, we establish a cutting-edge method for transfecting primary neurons and NSCs, named teleofection, by a two-step process to enhance the formation of biocompatible calcium phosphate (CaP) nanoparticles. Teleofection enables both nucleic acid and protein transfection into primary neurons and NSCs, eliminating the need for specialized skills and equipment. It can easily fine-tune transfection efficiency by adjusting the incubation time and nanoparticle quantity, catering to various experimental requirements. Teleofection’s versatility allows for the delivery of different cargos into the same cell culture, whether simultaneously or sequentially. This flexibility proves invaluable for long-term studies, enabling the monitoring of neural development and synapse plasticity. Moreover, teleofection ensures the consistent and robust expression of delivered genes, facilitating molecular and biochemical investigations. Teleofection represents a significant advancement in neurobiology, which has promise to transcend the limitations of current gene delivery methods. It offers a user-friendly, cost-effective, and reproducible approach for researchers, potentially revolutionizing our understanding of brain function and development.

## Introduction

Delivery of proteins and nucleic acids into cells, a technique called transfection, is a basic but critical strategy to study gene function. To date, numerous transfection methods have been developed based on physical, chemical, or biological mechanisms, each adapted to different experimental systems (Table S1).^1,2^ Over the past decades, considerable efforts were made in developing innovative materials and methods for cargo delivery in basic and translational research, but due to various biological limitations, such as high immunogenicity and cytotoxicity and low delivery efficiency, results have not fully met expectations.^2^ Since its development by F.L. Graham and A.J. van der Eb in 1973, the calcium phosphate (CaP) bioceramic system has been extensively utilized for *in vitro* gene transfection. This system offers excellent biological compatibility, biodegradability, cost-effectiveness, and ease of use, making it a widely adopted method to this day.^3^ The formation of the coprecipitation complex, facilitated by the interaction between positively charged calcium ions and negatively charged regions of the target molecules, is believed to be the key mechanism for uptake through endocytosis by specific target cells.^4−7^ Despite the advantages of the CaP-transfection method, however, the formation of optimal CaP nanoparticles is intricate and difficult to reproduce.^8^ Multiple parameters influence the crystallization and solubility of CaP nanoparticles, often resulting in suboptimal conditions (Table S2), leading to low transfection efficiency and high cytotoxicity in primary cultured cells.^9−11^ Efficient delivery of exogenous materials with high efficiency but low cytotoxicity into primary cells remains a limiting factor in many investigations.

Neural primary culture and neural stem cells (NSCs) are the most well-established and widely used models to study neuronal function, including neurotoxicity of drugs, neuronal plasticity, and the molecular bases of neuronal diseases. However, cultures of primary neurons are notoriously difficult to transfect, limiting their usability. Although numerous commercialized kits and experimental approaches have been developed to improve transfection efficiency, cytotoxicity, stress response induction, poor reproducibility, and excessive price remain limiting factors.^12−18^ CaP is arguably the simplest and cheapest reagent for cell transfection but suffers from about 1% poor efficiency and low cell viability (reagent consistency is critical for reproducibility) after transfection in primary neuron cultures.^12,13^ Many modified CaP-transfection protocols have been developed, but they can only be applied to very specific conditions because of the microscale size of CaP precipitates (uncontrolled growth of CaP nanoparticles), i.e., microisland and low-density cultures,^4,16,19−25^ which constrains their applicability (Table S3).

CaP nanoparticles tend to agglomerate or aggregate during synthesis rapidly, which can affect their dispersion and stability in biological solutions.^8^ Achieving the desired size and morphology of CaP nanoparticles is crucial for their specific applications. Moreover, while CaP nanoparticles are generally biocompatible, the introduction of other elements or compounds during synthesis may affect their biocompatibility.^8^ Ensuring that the final nanoparticles are safe for biological applications can be a challenge. To overcome these issues, it is crucial to control key reaction parameters such as mixing method, Ca/P ratio, temperature, pH, reaction time, and precursor concentrations. Here, we describe a two-step, simple method for preparing CaP nanoparticles, which resolves most of the limitations of the standard CaP methods. We named this method “teleofection” from the Greek *teleo* meaning “complete” for its greatly improved ability to deliver external materials intracellularly. Teleofection offers a distinct advantage by enabling efficient delivery of exogenous materials into cell lines, primary neurons, and NSCs with minimal cytotoxicity and exceptional reproducibility. This is in contrast to existing transfection methods, which, as far as our knowledge extends, are confined to nucleic acids, specific cell types, and limited applications. Notably, teleofection not only enhances transfection efficiency considerably and simply but also transfers nucleic acids and proteins into primary neurons, demonstrating its comprehensive capabilities for transfection. With highly reproducible and straightforward protocols, teleofection offers users a simpler and more cost-effective approach to transfect exogenous materials for various purposes, establishing itself as the method of choice across a multitude of applications, as exemplified in our demonstrations.

## Results and Discussion

### Generation of CaP Nanoparticles

**Figure 1:**
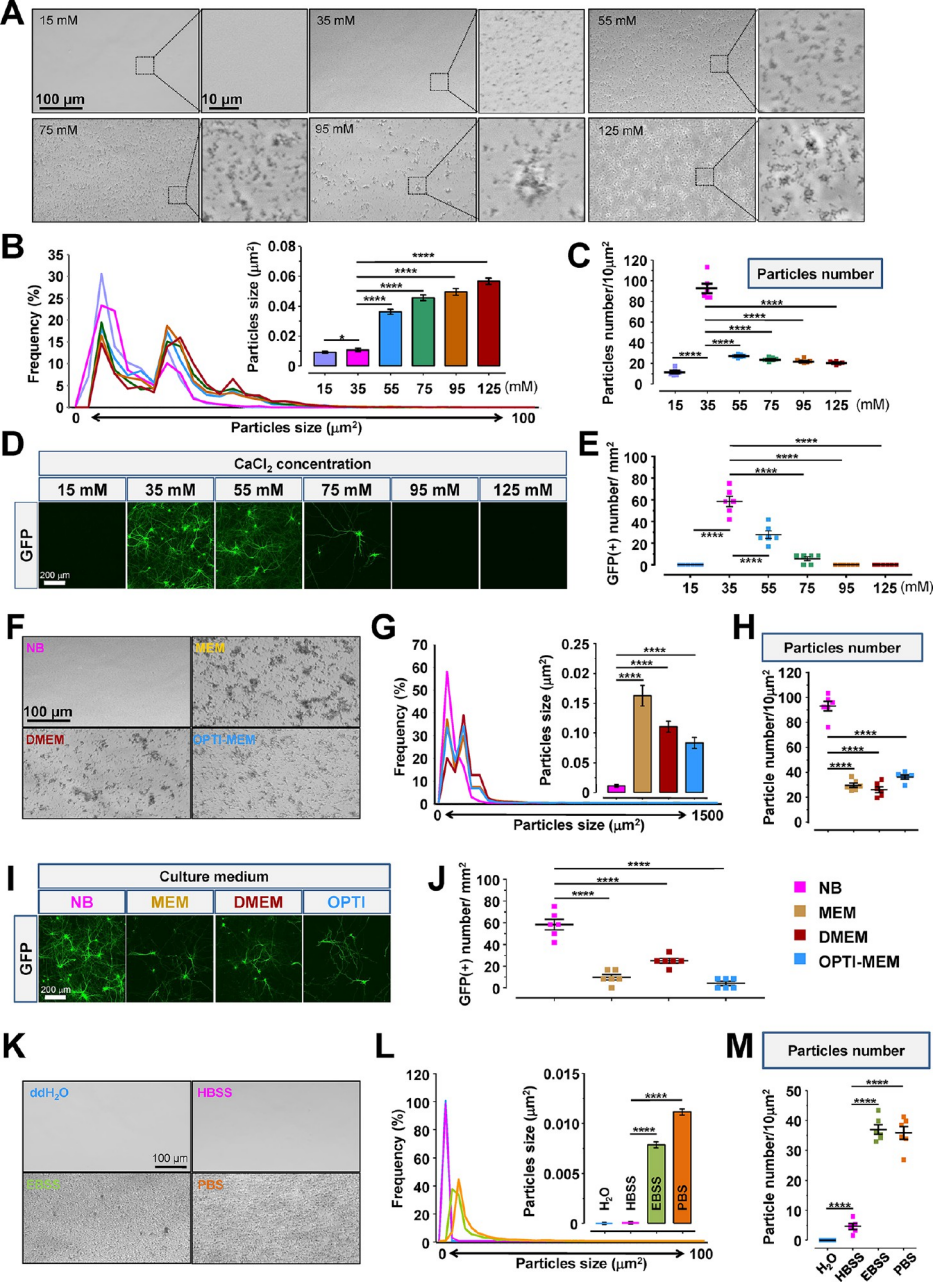
Calcium concentration and the types of medium determine the size and quantity of CaP nanoparticles. (A–C) Different CaCl_2_ concentrations caused various sizes and numbers of CaP particles. 35 mM CaCl_2_ shows a great number of CaP nanoparticles with relative smaller sizes. (D,E) The example images show the transfection efficiency of pLL3.7/*Syn-Gfp* under indicated CaCl_2_ concentration. GFP-positive neurons were in green. The quantitative data represent the numbers of GFP-positive cells under indicated CaCl_2_ concentration. (F–H) The size and quantity of CaP particles were changed by different media. Neurobasal (NB) medium has the most optimized condition to generate CaP nanoparticles. (I,J) The example images display the transfection efficiency of pLL3.7/*Syn-Gfp* under various media. GFP-positive neurons were in green. The quantitative data display the numbers of GFP-positive cells under indicated CaCl_2_ concentration. (K–M) The size and number of CaP nanoparticles were changed under different buffer systems. HBSS showed the most optimized condition to remove CaP nanoparticles. Over 1000 particles were analyzed from six different areas of three independent experiments. Statistic: one-way ANOVA (B, C, E, G, H, J, L, and M). Values represent the mean ± s.e.m. *P < 0.05, **P < 0.01, ***P < 0.001, ****P < 0.0001.

CaP nanoparticles can be synthesized by various methods, leading to different dimension, morphology, and architecture (Table S2).^8,26−29^ These nanoparticles exhibit sensitivity to even slight changes in temperature, pH, and salt concentrations, potentially causing undesirable heterogeneity and instability. However, this sensitivity to physicochemical conditions also presents an opportunity to manipulate these parameters and control the metastable amorphous state and size of CaP particles.^30,31^ In water at ambient temperature, saturated solutions of CaP nanoparticles tend to precipitate quickly and form crystalline particles.^32,33^ Lower crystallization rate conditions (e.g., temperature increase or reduction of calcium concentration) instead promote amorphous states and lead to a better-defined nanoparticle shape.^33,34^ Based on these observations and by optimizing the ratio between calcium and phosphate buffer, we generated small CaP nanoparticles (∼100 nm) with optimal delivery capacity and minimal cytotoxicity (Figure 1 and Supporting Information Figures S1 and S2).

Despite many suggestions from previous protocols,^4,16,19−25^ we discovered that calcium concentration and culture medium are critical factors that influence CaP nanoparticle formation and efficiency of transfection (Figure 1). In our system, CaP nanoparticles were created by mixing 50–70 mM calcium solution with 2× HEPES-buffered saline (HeBS) using either vortexing or pipetting, without the need for any further incubation, in just two simple steps (Supporting Information Figure S1 and Table S4). Although temperature, pH value, and reaction time have been reported as pivotal factors influencing the properties of the CaP particles,^8,11,30,32−35^ we find calcium concentration itself plays a foremost role in determining the size and characteristics of CaP particles. We observed that CaP nanoparticles were produced readily within a range of calcium concentrations at room temperature, with the ∼0.01 microm^2^ (∼100 nm in diameter) particles generated from a 35 mM calcium solution showing the best DNA delivery capability and lowest cytotoxicity under 5% CO_2_ and 37 °C (Figure 1A–E, Supporting Information Figure S2A, C, and E). Moreover, the CaP particle size and number were positively and negatively correlated with calcium concentrations (Figure 1A–C). Importantly, these ∼100 nm CaP nanoparticles are small enough to perform extensive Brownian motions (Videos S1–S3), which is significantly different from the lipofectamine-produced liposome and conventional CaP precipitates (precipitating on the bottom with slight shaking).^4,16,19−25^

Next, to define the effect of different media on CaP nanoparticle formation, several commonly used laboratory media were investigated (Figure 1F–J and Supporting Information Figure S2B). We discovered that the neurobasal (NB) medium not only promoted the formation of the smallest-sized and most numerous CaP nanoparticles but also stood out as the optimal medium for efficient DNA delivery, minimizing cytotoxicity (Figure 1F–J and Supporting Information Figure S2A, C, and F). Hank’s balanced salt solution (HBSS) was the most efficient buffer for nanoparticle washout after transfection (Figure 1K–M). The robustness of teleofection was further validated under various conditions, demonstrating that the buffer system consistently and reproducibly achieves effective transfection of primary neurons (Supporting Information Figure S2G–I).

### Transfection Efficiency Achieved in Primary Neurons through Teleofection

**Figure 2:**
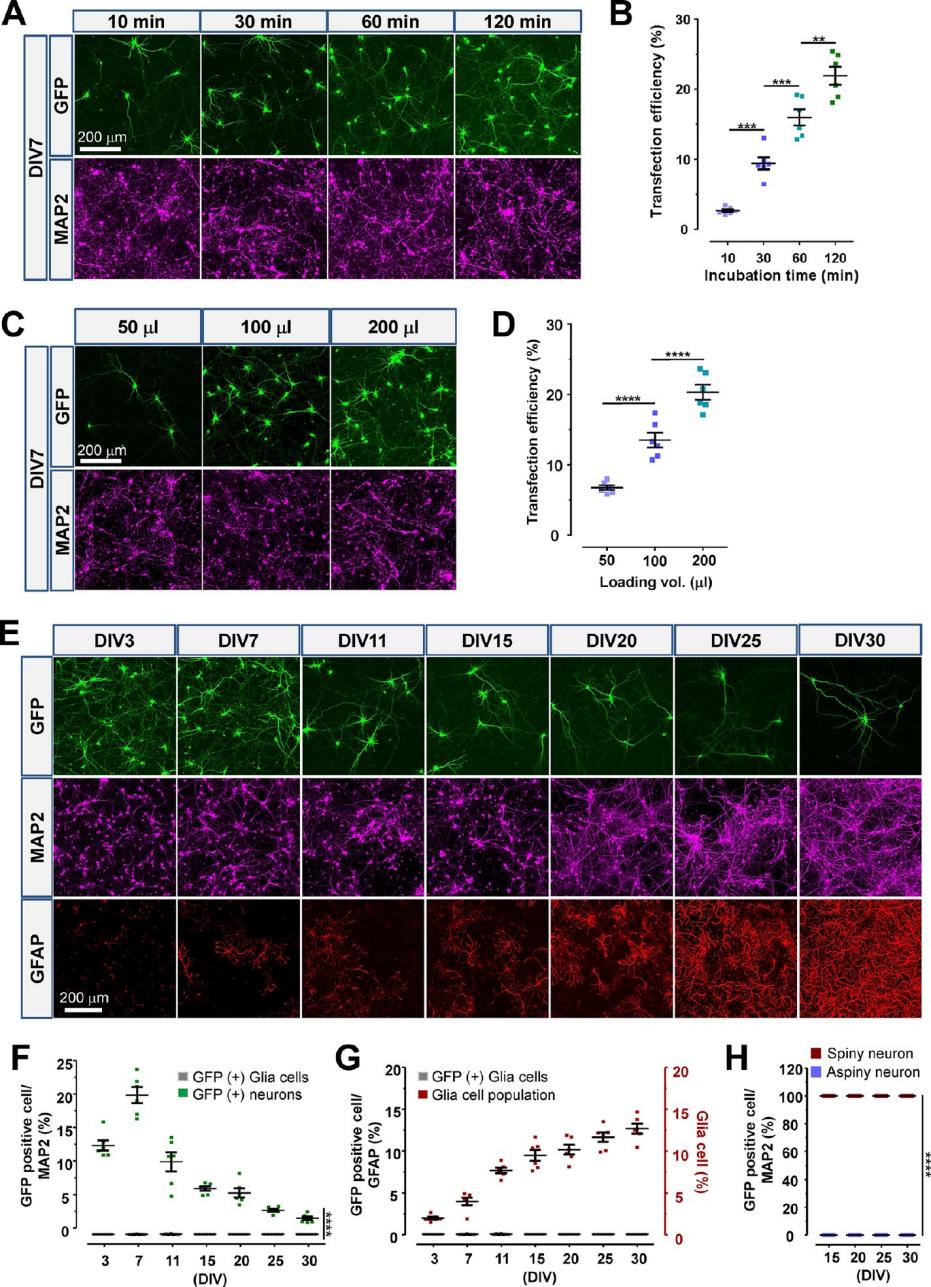
Teleofection delivers nucleic acid into primary neurons at different days *in vitro* (DIV) with desired efficiency. (A,B) The examples of images show the transfection efficiency of primary neurons under the indicated incubation times and CaP nanoparticle numbers. The transfected neurons were with GFP expression in green and neuronal marker MAP2 in magenta. (C,D) The quantitative data from A and B show the transfection efficiency of neurons under different transfection conditions. The transfection efficiency can be manipulated by change incubation time and loading volume of the teleofection mixture. (E) The representative images show the transfected neuron (GFP in green), glia cell marker (GFAP in red), and neuronal marker (MAP2 in magenta) at indicated time points in rat primary neuron culture. (F,G) The quantitative data of E show the transfection efficiency of neuron and glia at different DIV primary culture. (H) The quantitative data indicate the transfection efficiency of spiny and aspiny neurons under time-course manner. Data were analyzed from six different areas of three independent experiments. Statistic: one-way ANOVA (B and D) and two-way ANOVA (F and H). Values represent the mean ± s.e.m., *P < 0.05, **P < 0.01, ***P < 0.001, ****P < 0.0001.

To define the transfection efficiency of teleofection in primary neurons (MAP2 positive cells), time- and dose-dependent experiments were conducted. Transfection efficiency was positively correlated with the incubation time of the teleofection medium and the quantity of CaP nanoparticles (Figure 2A–D). Transfection efficiency increased gradually from 2.654 ± 0.204% to 21.89 ± 1.282% with an increase in the incubation time from 10 to 120 min (Figure 2A,B). An increase of CaP nanoparticles (from 50 to 200 μL) also significantly enhanced the transfection efficiency from 6.747 ± 0.331% to 20.37 ± 1.075% with 2 h of incubation (Figure 2C,D). We observed that teleofection demonstrates the capability to deliver plasmids with a molecular weight exceeding 10 kb while achieving the desired protein expression levels (Supporting Information Figure S3A). Notably, the disruption of importin-dependent nuclear transportation significantly reduced transfection efficiency and protein expression levels in primary neurons, highlighting the crucial role of importin-dependent nuclear transportation in teleofection-mediated gene expression (Supporting Information Figure S3B). Moreover, the application of endocytosis and importin inhibitors resulted in a significant reduction in the cytosolic and nuclear transportation of fluorescent dye-conjugated nucleic acid in primary neurons, respectively, which underscores the critical roles of dynamins and importins in DNA delivery to primary neurons (Supporting Information Figure S3C). We also examined the impact of CaP nanoparticles on the cellular physiology of primary neurons (Supporting Information Figure S3D–G). Despite causing a slight increase in the intracellular calcium concentration ([Ca2+]_ic_) and a minor insensitivity of sodium channels, neurons still exhibited a normal response to NMDA treatment after teleofection. Cell viability was assessed across various transfection strategies, revealing that teleofection exhibited minimal cytotoxicity comparable to the control group and demonstrated superior viability compared to other methods (Supporting Information Figure S4A–D). Because primary neurons display poor transfection efficiency after long-term culture, we sought to understand the transfection potency of teleofection on different days *in vitro* (DIV). Teleofection exhibited peak transfection efficiency at DIV7 (20.160 ± 1.187%) and started to reduce at DIV11 (9.7 ± 0.322%) gradually; yet it still maintained a notable potency of 1.293 ± 0.262% at DIV30 (Figure 2E,F). Moreover, teleofection showed similar work efficiency in rat (DIV7 = 20.160 ± 1.187%) and mouse (DIV7 = 20.910 ± 1.292%) primary neurons and had better transfection ability than the Lipofectamine 3000 (Lipo-3000) and conventional-CaP method (teleofection, DIV3 = 12.140 ± 0.761%, DIV7 = 20.160 ± 1.187%, DIV20 = 5.088 ± 0.679%; Lipo-3000, DIV3 = 1.093 ± 0.066%, DIV7 = 1.520 ± 0.060%, DIV20 = n.d.; conventional-CaP, DIV3 = 2.61% ± 0.518%, DIV7 = 3.635% ± 0.388%, DIV20 = n.d.) (Supporting Information Figure S4E,F). Upon further comparison with currently popular commercial kits, it was evident once again that teleofection exhibits lower cytotoxicity and higher transfection efficiency than these kits (Supporting Information Figure S4G–I). During long-term culture, the population of glial cells increased significantly, yet the numbers of GFP-positive glial cells remained considerably lower than those of primary neurons (Figure 2G). Interestingly, teleofection had a higher transfection efficiency in spiny neurons compared to aspiny neurons (Figure 2H). Additionally, we found that teleofection also achieved exceptional transfection efficiency in various cell lines, including HeLa cells and HEK293T cells (Supporting Information Figure S5). Altogether, teleofection demonstrated greater DNA transfection potency and reduced cytotoxicity for primary neurons. Moreover, teleofection selectively targets spiny neurons for DNA delivery, offering researchers the ability to control transfection efficiency by adjusting the quantity of CaP nanoparticles and transfection duration in a straightforward manner.

### Utilization of Teleofection for Observing the Spine–Axon Interaction and Neuronal Morphogenesis

**Figure 3:**
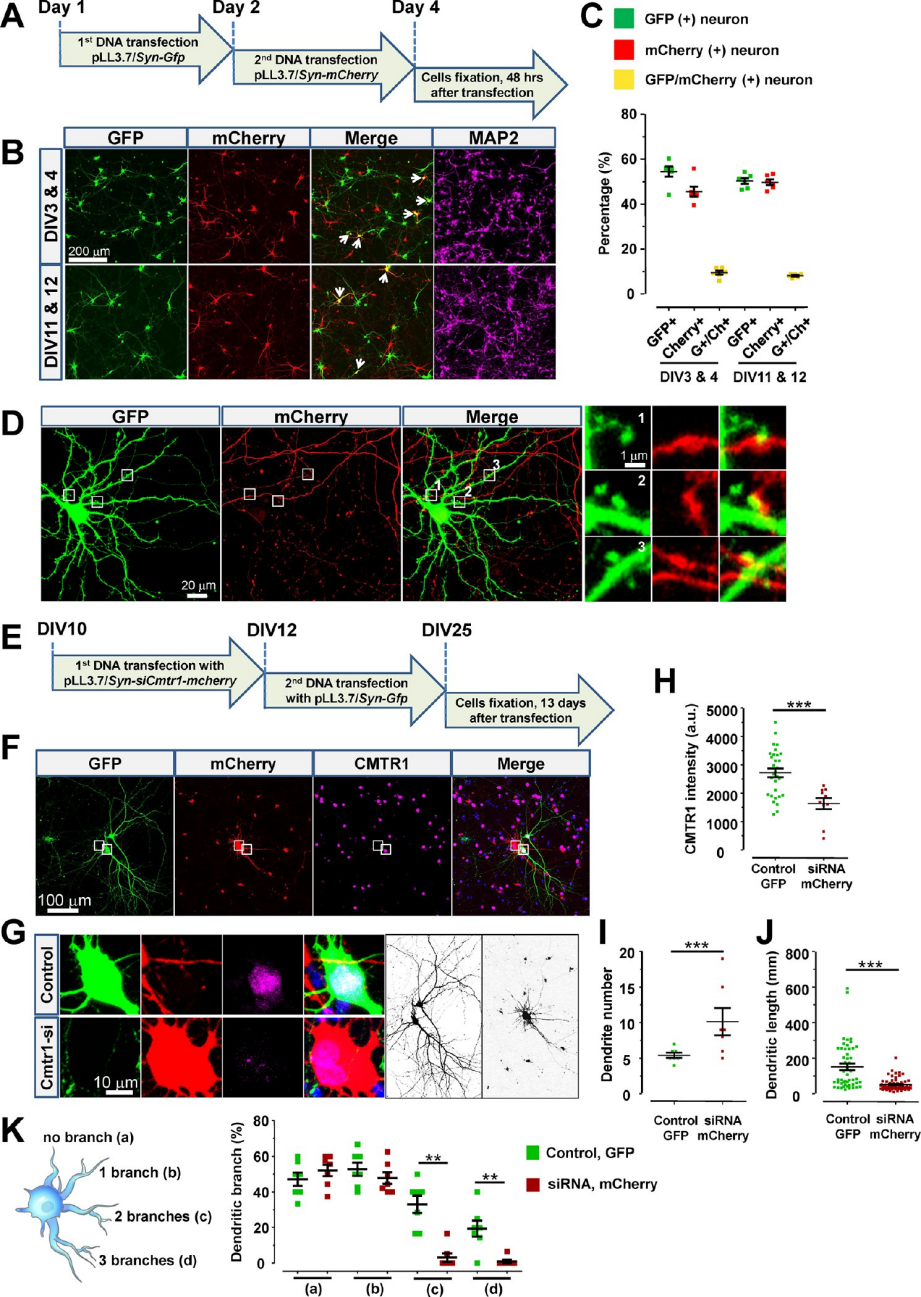
Teleofection enables versatile serial or cotransfection in primary neurons for diverse cellular biology research aims. (A,B) The flowchart shows the experiment design for serial transfection of indicated plasmids. The examples of images show the expression pattern of transfected neurons with GFP (green) or mCherry (red), and neurons were outlined by MAP2 (magenta). Under serial transfection, different nucleic acids can be uptaken by different neurons sequentially. The representative images show the neurons transfected with GFP followed by mCherry transfection at DIV10 and 11, respectively. The arrows indicate GFP and mCherry double positive neurons. (C) The quantitative data display that most of the neurons express only a single transfected gene, and only around 10% of neurons can uptake both transfected genes. (D) The representative images indicate that, based on serial-transfection stratagem, the synaptic region composed by two different neurons in green (dendritic spine) and red (axonal terminal) can be illustrated clearly. The thickness of a slice interval of a *Z*-stack is 0.5 μm. Also see Videos S9–S11. (E) The flowchart shows the experiment design for serial transfection of *Cmtr1* siRNA followed by pLL3.7/*Syn-Gfp* transfection at DIV7 and 10, respectively. The neurons were fixed for ICC at DIV20. (F) The representative images show the neuron-expressed GFP (green) and mCherry (red) as the control and *Cmtr1* knockdown group, respectively. The CMTR1 signal was displayed as magenta. (G) The high-magnification images from F show the details of transfected neurons. (H–J) The quantitative data from F and G represent CMTR1 intensity, dendritic number, and dendritic length in control and *Cmtr1* siRNA knockdown groups. (K) The cartoon displays the definition and the pattern diagram of the dendritic branch types for image analysis. The quantitative data display the percentage of dendritic branch types in control and *Cmtr1* siRNA-transfected neurons. Data were analyzed from at least six different areas of three independent experiments. Statistic: Student’s unpaired *t* test (H–K). Values represent the mean ± s.e.m., *P < 0.05, **P < 0.01, ***P < 0.001, ****P < 0.0001.

The plasticity and interconnection between dendritic spines and axons are crucial for maintaining functional neuronal circuitry and reflecting circuit rewiring. However, real-time monitoring of the dynamic spine–axon interaction remains a significant challenge in the field of neurobiology. Here, we present a simple protocol based on teleofection, providing real-time exploratory visualization of a heterotypic spine–axon or axon–axon interaction simultaneously (Figure 3A–D and Supporting Information Figure S6). The low cytotoxicity of teleofection means that serial transfection can be applied to cultured neurons for coexpression of multiple fluorescent tags or RNAi vectors. To illustrate this, after serial teleofection (Figure 3A) around 10% of neurons coexpressed both GFP and mCherry simultaneously, while nearly 90% of transfected neurons expressed either GFP or mCherry alone, consistently observed at different stages of neuronal development (Figure 3B,C). Despite the highly variable and dynamic nature of synaptic structures, the architecture and morphology of neurons can be unequivocally visualized through the expression of transfected reporter genes using teleofection (Supporting Information Figure S6 and Videos S4–S9). Serial teleofection allows axons and dendritic spines from heterotypic neurons to be distinguished by expressing different colors, allowing for real-time monitoring of pre- and postsynaptic interactions (Figure 3D and Videos S10–S12). In our system, the dendritic spine and axon manifested a highly dynamic movement and changed their morphology and connectivity frequently under normal conditions (Videos S10–S12). Additionally, teleofection enabled efficient delivery of various exogenous genes into the same culture cells at different incubation times, enabling the monitoring of morphological changes after specific gene knockdown, by cotransfecting a reporter gene (Supporting Information Figure S7).

Gene knockdown and overexpression are key methods to investigate the molecular function of target genes. Comparing control cells with gene-targeted cells is a crucial step in evaluating the biological significance of the specific gene. However, the current approach of examining cells in separate culture wells is limited. Serial teleofection however leads to heterogeneous population of neurons and allows the examination of morphological changes of control and gene-targeted primary neurons within the same culture well (Figure 3E–K and Supporting Information Figure S7E–I), as we illustrate next. *Cmtr1* is essential for neuromorphogenesis and brain development, and a deficiency of *Cmtr1* causes severe abnormality of the dendritic shaft;^36^ hence, we wondered whether this phenomenon could be reproduced by serial teleofection. The first teleofection was performed at DIV10 with the pLL3.7/*Syn-siCmtr1-mCherry* plasmid, followed by the second teleofection with pLL3.7/*Syn-Gfp* at DIV12 and fixation at DIV25 (Figure 3E). CMTR1 expression was significantly reduced in mCherry-positive neurons compared to the adjacent GFP-positive control (Figure 3F–H). Consistent with previous results,^36^ knockdown of CMTR1 caused severe abnormality of dendrites compared to adjacent GFP-positive control (Figure 3I–K). The applicability of serial teleofection was further validated by examining the morphological change of dendritic spines in *Cpeb2* knockdown neurons^37^ (Supporting Information Figure S7D). The significant downregulation of CPEB2 was detected in mCherry-positive neurons, exhibiting lower spine density compared to adjacent GFP-positive controls (Supporting Information Figure S7E–I). In conclusion, the low toxicity and high efficiency of teleofection enable either serial or cotransfection, providing a powerful and convenient way to study the function of candidate genes in primary neurons.

### Teleofection Enables Molecular and Biochemical Analyses of Primary Neurons

**Figure 4:**
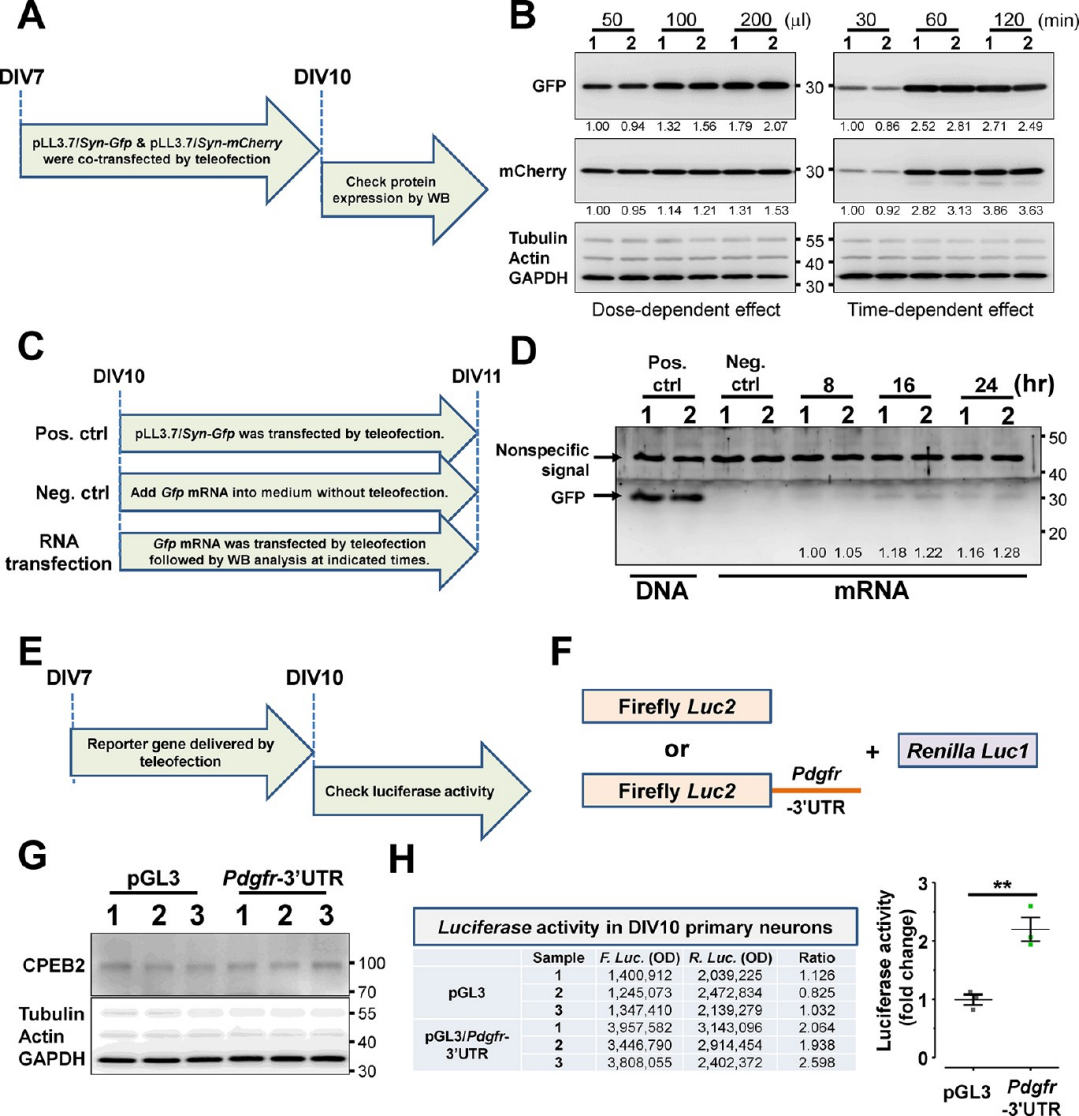
Teleofection is applicable for molecular and biochemical analysis. (A) The flowchart shows the experiment design for cotransfection of pLL3.7/*Syn-Gfp* and *-mCherry* at DIV7. Transfected neurons were harvested at DIV10 for Western blotting to detect the expression of GFP and mCherry. (B) The representative images show the protein expression level of delivery genes (*Gfp* and *mCherry*) under dose- and time-dependent manners in primary neurons. Two independent samples were displayed for each condition. Tubulin, actin, and GAPDH were detected as internal controls. (C) The flowchart shows the experiment design for *Gfp* mRNA transfection at DIV10, followed by sample collection at indicated times. (D) The representative image shows the expression level of GFP under time-dependent manners in primary neurons. (E) The flowchart indicates the experiment design for a dual-reporter assay in primary neurons. Firefly and Renilla *Luciferase* reporter plasmids were cotransfected into neurons at DIV7, and the reporter assay was performed at DIV10. (F) The expression of endogenous CPEB2 was detected by Western blotting. (G,H) The table represents the Luciferase activity of Firefly and Renilla in transfected neurons. The quantitative data were displayed as a dot plot graph in H. Data were analyzed from three independent experiments. Statistic: Student’s unpaired *t* test (H). Data were analyzed from three independent experiments. Values represent the mean ± s.e.m., *P < 0.05, **P < 0.01, ***P < 0.001, ****P < 0.0001.

The study of molecular and biochemical changes in primary neurons with genetic manipulation is a critical step in comprehending the mechanisms of target genes. Nevertheless, a persistently challenging aspect has been the low DNA transfection efficiency. Here, we present compelling evidence that teleofection serves as an optimal solution to address these longstanding concerns. Teleofection leads to stable expression of transfected DNA vectors, as we demonstrate by performing a Western blotting assay at DIV10, 3 days (DIV7) after teleofection (Figure 4A,B). The expression of GFP and mCherry was detected 3 days after teleofection, still showing a significant change of protein level under dose- and time-dependent manners (Figure 4B). There are cases when a translation from a template mRNA may be required, for example, when studying the stability of mRNA or the roles of 5′ or 3′ UTRs. We thus tested whether teleofection could be used to transfect primary neurons with mRNA. An equal amount of DNA or mRNA was delivered by teleofection, and the GFP expression level was analyzed by Western blotting, which showed a peak level after 16 h of RNA delivery (Figure 4C,D).

Additionally, the reporter assay is another valuable tool for studying transcriptional and translational regulatory mechanisms, but it still has difficulty conducting in primary neurons. The 3′-UTR of *Pdgfr*, a known target positively regulated by CPEB2,^38^ was incorporated following the Firefly *Photinus pyralis luciferase* gene (*Luc2*) for a reporter assay after teleofection. Plasmid-expressed *Luc2-Pdgfr*-3′-UTR (reporter) and the *Renilla luciferase* gene (*Luc1*; internal control) were cotransfected into primary neurons at DIV7 by teleofection, and the reporter activity was examined at DIV10 (Figure 4E,F). Over a million levels of luminescence intensity from Firefly and Renilla luciferase were detected, showing a similar expression tendency as seen in a previous study (Figure 4G,H). These findings collectively suggest that teleofection is an effective approach for delivering both DNA and RNA into primary neurons, enabling further molecular and biochemical investigations.

### Flexibility of Teleofection Allows Delivery of Functional Proteins into Primary Neurons

**Figure 5:**
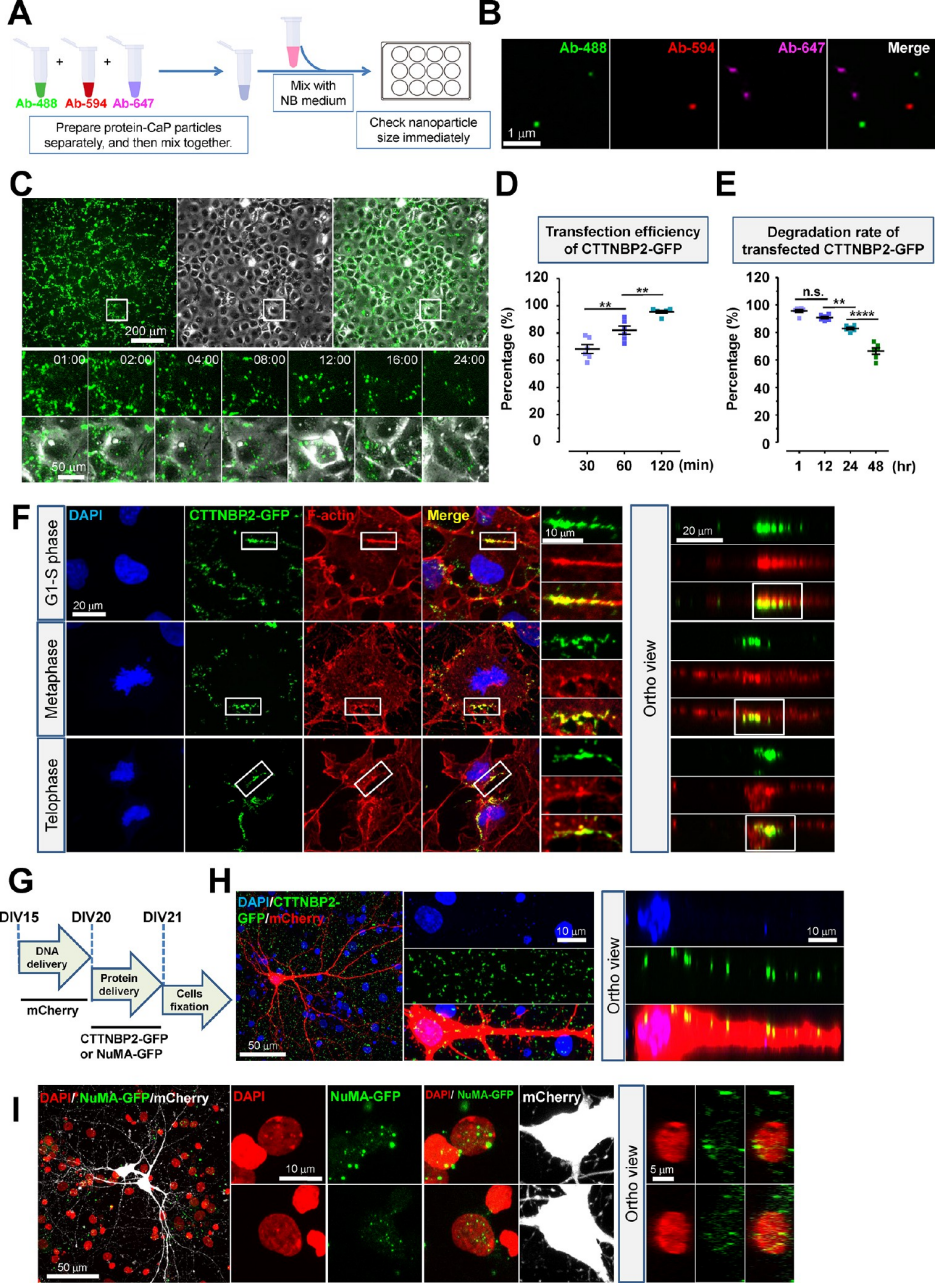
Teleofection is a stratagem for protein delivery in cell lines and primary neuron culture. (A) The flowchart shows the experiment design to generate different CaP nanoparticles with distinct proteins for the cell delivery. Also see Supporting Information Figure S8 for the stratagem of protein cotransfection by teleofection. (B) The representative images showcase nanoparticles containing various fluorescent dye-conjugated antimouse antibodies (Ab), showing green for Ab-488, red for Ab-594, and magenta for Ab-647. (C) The representative images show the global amount of CTTNBP2-GFP (green) in COS7 cells after protein transfection by teleofection. The high magnification displays the changes of CTTNBP2-GFP in a COS7 cell under time-dependent manner. The bright field displays the details of cell morphology. (D–E) The dot plot graphs represent the quantitative data of transfection efficiency and the degradation rate of CTTNBP2-GFP in COS7 cells. (F) The example of images indicates the distribution of CTTNBP2-GFP (green) in COS7 cells under different cell cycle stages. Nuclei and stress fibers were outlined by DAPI (blue) and phalloidin (red), respectively. The colocalization of CTTNBP2-GFP and stress fiber was displayed as yellow. (G) The flowchart indicates the experiment design to deliver reporter gene and protein sequentially in primary neuron culture. (H) The representative images show the distribution of CTTNBP2-GFP (green) in primary neurons after 24 h of transfection. The low-power field displays the global view of the primary neuron labeled by mCherry (red). Nuclei were labeled by DAPI in blue. The high magnification reveals the detailed distribution of CTTNBP2-GFP in the dendritic shaft. The colocalization of CTTNBP2-GFP and mCherry was manifested as yellow. (I) The representative images show the distribution of NuMA-C-GFP (green) in primary neurons after 24 h of teleofection. The low-power field displays the global view of primary neurons labeled by mCherry (white); nuclei were displayed as red by DAPI. The high magnification shows the distribution of NuMA-C-GFP in nuclei; the colocalization of NuMA-C-GFP and the nucleus was manifested as yellow. Data were analyzed from six different areas of three independent experiments. Statistic: One-way ANOVA (D and E). Values represent the mean ± s.e.m., *P < 0.05, **P < 0.01, ***P < 0.001, ****P < 0.0001.

Since CaP nanoparticles have been demonstrated to form the complex with proteins in serum, named calciprotein particles (CPPs), which are composed of CaP crystals and mineral binding proteins such as Fetuin-A,^39,40^ we wonder whether teleofection can generate CPPs for protein delivery in primary neurons. To test this hypothesis, fluorescent dye-conjugated mouse IgGs were employed to generate CPPs by teleofection (Figure 5A). We found that teleofection can generate CPPs containing various proteins at nanoscale size (Figure 5B). Different protocols allowed for the incorporation of single or multiple fluorescent proteins into CPPs for diverse applications (Supporting Information Figures 8A–D), which exhibited efficient delivery of CPPs into cultured cells (Supporting Information Figure S8E–H). We further characterized the transfection ability of teleofection-produced CPPs by using GFP-tagged Cortactin-binding protein 2 (CTTNBP2), an autism spectrum disorder (ASD)-associated protein, that controls neuronal morphogenesis through a liquid-to-gel phase transition pathway.^41−43^ CTTNBP2-GFP-CPPs exhibited efficiency comparable to that of fluorescent dye-conjugated IgGs when delivered into COS7 cells (Figure 5C,D). Despite protein degradation observed after delivery, over 65% of cells retained GFP-positive signal at 48 h postteleofection (Figure 5E). CTTNBP2-GFP demonstrated biological activity, exhibiting colocalization patterns with actin-stress fibers and contractile rings (Figure 5F).^43,44^ Notably, CTTNBP2-GFP could also be transfected into primary neurons using teleofection, resulting in a punctate morphology along dendritic shafts and soma, consistent with previous findings (Figure 5G,H).^44^ Additionally, NuMA, a nuclear protein associated with the mitotic apparatus,^45^ was conducted to investigate the nuclear distribution of teleofection-produced CPPs. The NuMA tail domain tagged with GFP (NuMA-C-GFP) was successfully transported into nuclei of primary neurons at 24 h postteleofection (Figure 5I). Altogether, teleofection offers an innovative method for delivering exogenous proteins into primary neurons while preserving their biological activity, which enables us to monitor the interaction and subcellular redistribution of target proteins.

### Teleofection Facilitates the Delivery of Nucleic Acids and Functional Proteins into Primary NSCs

**Figure 6:**
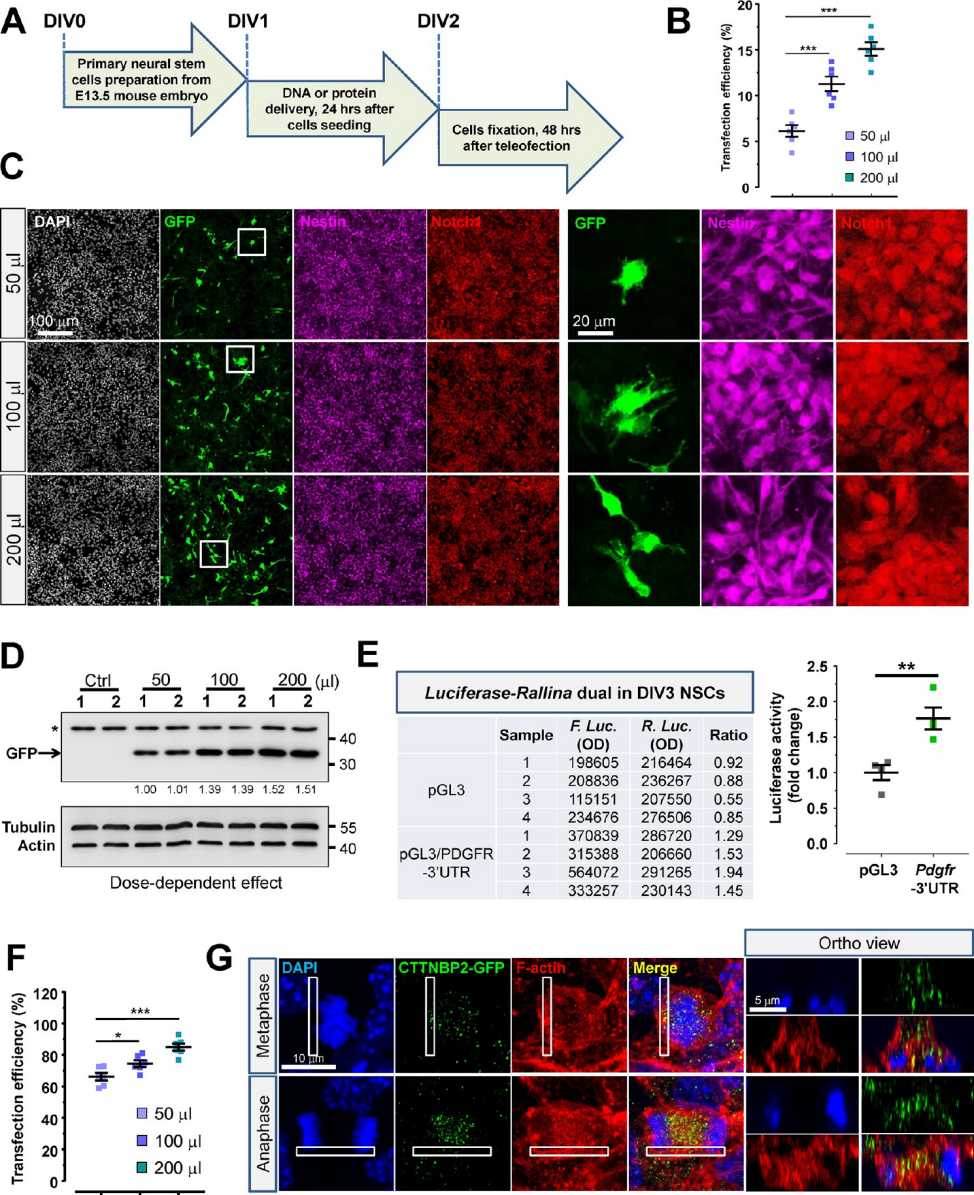
Teleofection introduces an innovative method for transporting both nucleic acids and proteins into NSCs. (A) The flowchart shows the experiment design to deliver nucleic acid into NSCs by teleofection. (B,C) The dot plot graph represents the quantitative data of transfection efficiency of pLL3.7/*Syn-Gfp* in NSCs. The representative images show the population of GFP-positive cells after teleofection. The high-power field exhibits the coexpression of NSC markers, Nestin and Notch1, in GFP-positive cells. (D) The example images show the expression level of GFP under dose-dependent manners by Western blotting after teleofection in NSCs. (E) The table represents the activity of Firefly and *Rellina* Luciferase in transfected NSCs. The quantitative data were displayed as a dot plot graph. Data were analyzed from four independent experiments. (F) The dot plot graph displays the transfection efficiency of CTTNBP2-GFP in NSCs by teleofection with a dose-dependent manner. (G) The representative images show the distribution of CTTNBP2-GFP (green) in NSCs after 24 h of teleofection. Nuclei and F-actin were labeled by DAPI and phalloidin in blue and red, respectively. The colocalization of CTTNBP2-GFP and F-actin was manifested as yellow. The orthogonal view of white squares from NSCs provides a comprehensive understanding of the relative distribution of CTTNBP2-GFP and F-actin from multiple angles. Statistic: One-way ANOVA (B and F). Student’s unpaired *t* test (E). Values represent the mean ± s.e.m., *P < 0.05, **P < 0.01, ***P < 0.001, ****P < 0.0001.

Extensive research has been conducted on neural stem cells (NSCs) due to their therapeutic potential. Nevertheless, delivering exogenous materials into NSCs for studying molecular mechanisms remains a formidable challenge, primarily due to their inherently low transfection efficiency.^46−48^ Despite the development of several methods aimed at enhancing transfection rates,^48−52^ these approaches are often constrained by factors such as reduced cell viability, heightened cytotoxicity, biosafety concerns, and elevated costs. Since teleofection has been demonstrated to effectively deliver nucleic acids and proteins in primary neurons, we speculate that teleofection might potentially exhibit similar capabilities in NSCs. To reveal the transfection potency of teleofection in NSCs, mouse embryos at embryonic day (E)13.5–14 were sacrificed for pluripotent NSC preparation, and dose-dependent experiments were conducted 24 h after cell seeding (Figure 6A), showing around 95% cells with NSC markers (Nestin and Notch1) (Supporting Information Figure S9A and C). Transfection efficiency exhibited a positive correlation with the loading volume of CaP nanoparticles (Figure 6B,C) and got higher gradually from 6.133 ± 1.521% to 15.07 ± 1.768% when the numbers of nanoparticles increase, which expressed greater gene delivery capability than previous methodologies (Supporting Information Figure S9B–C). GFP overexpression was evident in immunoblotting assays, displaying a dose-dependent response consistent with immunostaining results (Figure 6B–D). Importantly, only p*CMV-Gfp*, but not pLL3.7/*Syn-Gfp*, was detected in NSCs, suggesting the presence of NSC-specific characteristics in the system (Supporting Information Figure S9D).

Furthermore, dual-reporter assays were performed to evaluate the applicability of teleofection in studying transcriptional and translational regulatory mechanisms in NSCs. Cotransfection of Firefly and Renilla luciferase into NSCs resulted in robust luminescence signals, with both luciferase’s activity showing high-intensity levels in the hundreds of thousands (Figure 6E). These findings collectively demonstrate that teleofection is a highly effective method for delivering nucleic acids into NSCs, facilitating extensive molecular and biochemical investigations. We further investigated the potential of protein transfection into NSCs using CTTNBP2-GFP through teleofection. The transfection efficiency showed a positive correlation with the amount of CaP nanoparticles loaded, gradually increasing from 66.170 ± 2.389% to 85.018 ± 2.269% 24 h after teleofection (Figure 6F). Even at 48 h post-teleofection, over 55.375 ± 4.508% of cells still displayed the CTTNBP2-GFP signal, indicating the long-lasting presence of transfected protein in NSCs (Supporting Information Figure S9E,F). Importantly, we observed that transfected CTTNBP2-GFP exhibited a colocalization pattern with actin stress fibers, suggesting the sustained normal biological function of CTTNBP2-GFP after teleofection (Figure 6G). To further understand the impact of teleofection on cell division and proliferation, the mitotic index and DNA content change were explored by immunocytochemistry (ICC) and flow cytometry, indicating no significant influence of CaP nanoparticles on these two factors in NSCs and 293T cells (Supporting Information Figure S9G–I). Overall, teleofection also manifests the higher delivery ability of nucleic acids and proteins in the primary NSC system, which could facilitate a broader comprehension, allowing for the expansion of molecular and cellular analysis.

## Conclusions

The efforts to optimize the transfection efficiency of primary neurons have been illustrated over the decades. Yet, a method with seamless merging simplicity, low cytotoxicity, cost-effectiveness, and impeccable reproducibility for both primary neurons and NSC transfection has remained elusive. We now provide comprehensive guidelines for achieving straightforward, low cytotoxicity, and high cost-effective transfection. Teleofection caters to both primary neurons and NSCs, ensuring reproducibility across diverse analytical approaches. Noticeably, teleofection-generated CaP nanoparticles form intricate complexes for delivering not only nucleic acids but also proteins. We highlight and compare commonly used methods with high transfection efficiencies to teleofection (Table S3). The discussion evaluates their advantages and disadvantages, encompassing transfection efficiency, expression levels, cell viability, complexity, reproducibility, cost, and applicability.

With high efficiency, virus transfection is commonly employed to deliver nucleic acids into neurons. Despite the fact that most recombinant viral vectors currently in use are replication incompetent and ensure a comparative level of safety, they still require additional safety measures and biosafety level 2 facilities (Table S1). Moreover, the substantial disadvantage of infection-induced antiviral intrinsic immunity may cause unexpected responses.^53^ To avoid these drawbacks, chemical and physical methods provide alternatives, with physical methods offering near 100% efficiency but facing equipment costs and cell damage challenges.^54^ Chemical-based methods, like CaP and cationic lipids (e.g., Lipofectamine), aim to overcome these issues. Lipofectamine achieves high delivery in cell lines (>85%) but only ∼1% in primary neurons due to the technique and reagent consistency.^13,55,56^ Using MAP2 labeling, Lipo-3000, an advanced lipid-nanoparticle technology, achieved 1–1.5% and 0.7% transfection efficiency in primary neurons and NSCs, respectively, showing a significantly lower transfection rate than teleofection (Supporting Information Figure S4F). Although Lipo-3000 and conventional CaP methods are less toxic in cell lines, their toxicity was higher in primary neurons compared to that with teleofection (Supporting Information Figure S4A–D). With its enhanced transfection efficiency, teleofection offers a means to conduct serial and simultaneous transfections for pre- and postsynaptic neurons, as well as molecular and biochemical analyses—tasks that are challenging to perform using the commercial kits and conventional CaP method.

Particle size significantly impacts nanoparticle stability, biodistribution, and cellular uptake.^57,58^ Conventional CaP methods often recommend dropwise aeration and mixing of DNA-Ca^2+^ solution with 2× HeBS buffer using gentle shaking to generate smaller particles.^20−23^ However, this approach’s reproducibility is inconsistent, leading to larger CaP precipitates that can hinder transfection efficiency and cell viability.^56^ Additionally, conventional protocols use acidified media under 10% CO_2_ incubation to dissolve CaP precipitates,^20,23,59^ which could damage primary neurons and cause cell death.^60^ Teleofection provides the two-step simple protocol to generate CaP nanoparticles with the greatest reliability in cell lines, primary neurons, and NSCs. Critically, we improved the manner to remove CaP nanoparticles by simply substituting the conventional wash buffer with HBSS without additional acidification, effectively cleaning the CaP nanoparticles and reducing cytotoxicity. Moreover, nanoparticle quantity greatly influences transfection efficiency.^61^ Lipo-3000 yields uniform-sized nanoparticles, while teleofection produces significantly higher nanoparticle quantities compared to Lipo-3000. Although increased liposome numbers can improve the transfection efficiency, it is essential to manage the associated cytotoxicity, which remains a major concern.^62,63^

Instead of DNA transfection, the use of mRNA in transfection not only allows for tracking subcellular processes but also serves as a safer alternative for transient gene expression without genetic modification.^64^ Compared to the corresponding DNA plasmid, the smaller size of mRNA may facilitate its transfer into the cell.^65^ Despite a short half-life, transfected mRNAs generate proteins in a brief period, and their protein products can persist for an extended period.^65^ Additionally, mRNA translation occurring exclusively in the cytosol bypasses the need for nuclear entry, potentially making transfection more efficient for postmitotic cells.^66^ To ensure the transfection efficiency of mRNA, various delivery strategies have been employed (Table S6). Despite higher efficiency, physical methods, such as electroporation, face technical challenges for *in vivo* applications. Therefore, chemical carriers, including lipids and organic polymers, have been advanced further for mRNA transfection. A handful of modifications have been applied to enhance transfection efficiency, including mRNA modification, the addition of chemical agents, and the development of synthetic nanoparticles.^64,67^ In this study, we initially employed teleofection for the transfection of mRNA into neurons. Despite the lower efficiency than DNA and protein delivery, the application on immunoblotting is workable, indicating the potential application of CaP-based mRNA delivery in a fundamental study under further improvement.

When choosing a method, it is important to weigh the pros and cons against the experimental goals, prioritizing high transfection efficiency, low cell toxicity, physiological integrity, user-friendliness, and reproducibility. Teleofection provides most of these desired features for applications in cell lines, primary neurons, and NSCs; however, some challenges in its application remain to be tackled. In terms of protein delivery, teleofection demonstrated over 80% transfection efficiency in primary neurons and NSCs (Figures 5D,E and 6F), but the existence of transfected proteins decreased to around 60% after 48 h, suggesting the involvement of an intracellular system in the elimination of these exogenous materials is the major challenge. As exogenous materials often utilize endocytosis pathways (pinocytosis and phagocytosis) for cellular uptake and are then eliminated frequently,^4−7^ successful transfection and prevention of introduced material degradation depend on proper intracellular distribution. The endosomal barrier remains a major bottleneck for effective nonviral nanocarrier delivery. Despite attempts to mitigate endosome acidification and enhance endosomal escape/lysis, issues such as nonspecific blockage-induced cytotoxicity and cellular abnormalities persist.^68^ Endosome lysis is hypothesized to occur rarely after endocytosis, allowing the release of endocytosed materials before fusion with lysosomes. Optimizing this process could enhance the delivery efficiency of exogenous materials. The “proton sponge effect” suggests that the weakly basic molecule can induce endosome bursting, facilitating the release of exogenous materials before fusion with lysosomes.^68,69^ Thus, leveraging the proton sponge effect might offer a potential strategy to improve cargo delivery and enable efficient transfection without additional cell-damaging agents, which will be particularly helpful in moving the nanomedicine field forward.

## Experimental Section

### Chemicals and Reagents

The chemicals used in this study include β-mercaptoethanol (Sigma, 60–24–2), CaCl_2_ (Sigma, C7902), ethylenediaminetetraacetic acid (Sigma, E9884), glucose (Sigma, G8270), glutamine (Sigma, G3126), glutamate (Sigma, 49621), HCl (Sigma, 320331), HEPES (Sigma, H3375), KCl (Sigma, P3911), l-cysteine (Sigma, 168149), MgCl_2_ (Sigma, M8266), NaCl (Sigma, S9888), Na_2_HPO_4_ (Sigma, S9763), NaHCO_3_ (Sigma, S5761), NaOH (Sigma, S8045), paraformaldehyde (Sigma, 30525–89–4), tris-base (Sigma, T1503), papain (Sigma, P4762), protease inhibitor cocktail (Roche, 04693116001), phosphoSTOP (Roche, 4906837001), DNase I (Roche, 11284932001), 2.5% trypsin no phenol red (ThermoFisher, 15090046), B-27 Supplement (Gibco, A1486701), FBS (Gibco, F244), antibiotic/antimycotic (Gibco, 15240–062), and poly-l-lysine (Sigma, P2636). Fura-2, AM, cell permeant (Thermo Fisher, F1221) was used for living-cell calcium image recording. The media and buffer systems are neurobasal medium (Gibco, 21103049), DMEM (Gibco, 11995054), MEM (Gibco, 12492013), OPTI-MEM (Gibco, 11058021), EBSS (Gibco, 14155063), and HBSS (Gibco, 14175095). ProLong antifade (Molecular Probe, P36930), 4′,6-diamidino-2-phenylindole, and dihydrochloride/DAPI (Invitrogen, D21490) reagents are used for immunostaining. The Dual-Luciferase Reporter Assay System (Promega, PAN1620) and PrestoBlue Cell Viability Reagent (Thermo Fisher, A13261) are for the reporter assay and cell viability assay. Antibodies used in detecting specific proteins are GAPDH (Santa Cruz, sc-25778), β-actin (Sigma-Aldrich, A5441), α-tubulin (Sigma-Aldrich, T5168), CMTR1 (Bethyl, A300–304A), GFAP (Millipore, MAB360), MAP2 (Novus, NB300–213), Nestin (BD Biosciences, BD561230), Notch1 (Cell Signaling, 3608s), Cleaved Caspase-3/Asp175 (Cell Signaling, 9661), GFP (Clontech, 632381), and mCherry (Abcam, ab167453). For immunofluorescence, AlexaFluor 488, 594, or 647-conjugated secondary antibodies were from Invitrogen. For immunoblot assay, horseradish peroxidase (HRP)-conjugated secondary antibodies were from Invitrogen. The commercial transfection kits includes Cytofect Neuron Transfection Kit (Cell Applications, TF886 KS), DreamFect Transfection Reagent (OZBiosciences, DF40500), GenJet In Vitro DNA Transfection Reagent (SignaGen Laboratories, SL100488), GenMute Transfection Reagent (SignaGen Laboratories, SL100568), Lipofectamine 3000 Transfection Reagent (Thermo Fisher, L3000001), Lipofectamine RNAiMAX Transfection Reagent (Thermo Fisher, 13778075), Nupherin Transfection Reagent (Zneo, BML-SE225-0075), PolyJet Transfection Reagent (SignaGen Laboratories, SL100688), TransIT-X2 Dynamic Delivery System (Mirus Bio, MIR6003), TransIT-LT1 Transfection Reagent (Mirus Bio, MIR2304), and TurboFect Transfection Reagent (Thermo Fisher, R0531). DNA labeling was processed by using a Label IT Nucleic Acid Labeling Kit, MFP488 (Mirusbio, MIR 7100).

### Experimental Animals

All rats and mice used for experiments were standard SD and C57BL/6 strain. Rodents were maintained in a 12 h light/12 h dark cycle (LD) with food and water *ad libitum*. Before sacrifice, mice were anesthetized with Avertin (0.25 mg/g body weight). All animal experiments were approved by the animal experimentation committee of Taipei Medical University and Academia Sinica and performed in accordance with the guidelines of the institutional committee for the use of animals for research. The different groups were housed together in the same cages in all animal experiments to prevent different environmentally caused effects.

### Plasmid Construction^36,38,70^

The oligonucleotides containing short hairpin RNA (shRNA) sequences, targeted against rat *Cmtr1*, *Cpeb2*, or a nontarget control, were constructed into pLentiLox (LL)3.7/*Syn* plasmid with *Hpa*I and XhoI restriction enzymes cutting sites for cloning. The (GCAGCCCTGCTCTGATGGT) shRNA clone against *Cmtr1* and (GCAGAAAGCAAGTCCTATT) shRNA clone against *Cpeb2* were designed for gene targeting. For a reporter assay, mouse PDGFRα 3′-UTR was PCR-amplified from lung cDNA for further construction. The amplified DNA fragment was cloned to the pGL3 promoter plasmid (Promega) using XbaI and *Sal*I cloning sites. The resulting plasmid was digested with XbaI and self-ligated to generate the 3′UTR 1-kb construct. The plasmid pT7-EGFP-N1 used for *in vitro* transcription was constructed by linearization of pEGFP-N1 (Clontech) with NheI and BgIII followed by ligation with the annealed oligonucleotides of the T7 promoter (5′-CTAGTAATACGACTCATATAGGGA-3′ and 5′-GATCTCCCTATATGAGTCGTATTA-3′). For pLL3.7/*Syn*-*mCherry* and pLL3.7/*Syn*-*Gfp*, the cytomegalovirus (CMV) promoter in the pLL3.7 plasmid was placed with *mCherry* to produce pLL3.7-Syn/*mCherry*. For pGW1-HA-Sbf1, *Sbf1* was amplified by specific primers, forward 5′-GCTGGTCGACATGGCGCGGCTCGCGGAC-3′, and reverse 5′-GGAATTCGGCATCCGACAGGCAGC-3′, with flanking of *Sal*I and *Eco*RI restriction enzyme cutting sites. The plasmids used in this study were listed in Table S5.

### Neuron Transfection Protocols

For primary neurons, the culture medium needs to have an appropriate volume in each well before performing teleofection (see Table S4 for details). Teleofection can be conducted from DIV0 to any culture day. For nucleic acid delivery, the teleofection mixture was prepared by mixing 2.5–3.5 μL of 2 M CaCl_2_, 1 μg of DNA, and appropriate H_2_O with a final volume of 100 μL for one well (12-well plate). The adjustment of the CaCl_2_ concentration is mainly dependent on the pH value of the culture medium. Acidification occurs gradually during long-term culture to cause higher solubility of CaP nanoparticles, so slightly increasing the nanoparticle size by changing CaCl_2_ concentration will improve transfection efficiency in the long-term culture system. The CaP nanoparticle–cargo mixture was obtained by adding 100 μL of 2× HeBS (12 mM Dextrose, 50 mM HEPES, 10 mM KCl, 280 mM NaCl, 1.5 mM Na_2_HPO_4_·2H_2_O, pH 7.05 with 10 N NaOH adjustment) dropwise (about 10 μL each time) to a cargo and CaCl_2_ mixture with a continuously gentle vortex (Vortex Mixer/GENIE2, power 4–5), and further incubation is unnecessary. Then, 400 μL of NB medium was taken from culture cells and added into the CaP nanoparticle–cargo mixture with 10-times pipetting without a vortex to get a teleofection medium. Before the teleofection medium is put into culture neurons, it is important to transfer and keep the rest of the culture medium for further incubation after teleofection. Teleofection medium was dropped into culture neurons gently, and neurons were then incubated at 37 °C, in a 5% CO_2_ incubator for 10–120 min. Incubation time is flexible and dependent on the purpose of the experiment; the longer incubation time shows higher transfection efficiency. The 2 h incubation displayed great transfection efficiency and has similar result as compared with longer incubation. After appropriate incubation, the teleofection medium was removed, and the rest of the CaP nanoparticles were washed out by warm HBSS three times (just add and remove HBSS without extra incubation). The conditioned (original) culture medium was put back into cultured neurons for subsequent incubation. For delivery of proteins into primary neurons, the protocol is similar to the delivery of nucleic acids, but 1 μg of DNA was replaced by 1 to 10 μM proteins. The incubation time of protein delivery is also dependent on the purpose of the experiment. For the cotransfection of multiple nucleic acids or proteins, the only difference is that target genes or proteins were added simultaneously into CaCl_2_ solution to generate a teleofection mixture for further process. For the serial transfection of different target materials, the delivery protocol for each cargo is the same as the above illustration. Notably, the optimal condition is to deliver the second cargo 1 day after the first teleofection, which will prevent the expression of two target genes in the same neuron. For the methods of transfection from the commercial kits and conventional CaP, we followed the protocols illustrated in the companies’ user manuals and Jiang’s report,^20^ respectively.

### Cell Lines, Neural Primary Culture, and Stem Cell Culture^71,72^

Primary neuron culture was processed according to previous studies. Rat or mouse cortices isolated from 18th ∼ 19th embryonic day brains were dissected into small pieces (1–2 mm^3^) and then washed with Hank’s balanced salt solution without calcium and magnesium (HBSS/no Ca^2+^–Mg^2+^) to remove contaminations. Tissues then digested in papain solution (0.6 mg/mL papain, 0.6 mg/mL DNase I, 0.2 mg/mL l-cysteine, 1.5 mM CaCl_2_, and 0.5 mM EDTA in HBSS) at 37 °C for 20–30 min with gentle shaking, followed by the 10% horse serum in NB medium treatment to stop the enzymatic reaction. Suspension neurons were obtained by 20–30 times gentle pipetting with a 10 mL plastic pipet (Thermo Scientific Nunc, 170536N). A 40 μm^2^ cell strainer was conducted to remove undigested tissues, and suspended cells were collected by centrifugation at room temperature (RT) with 1000*g* for 5 min. The cells were resuspended in NB medium containing 0.5 mM glutamine, 12.5 μM glutamate, 1× antibiotic–antimycotic, and B27 supplement. A cell density of 3 × 10^5^ cells/well was added into the PLL-coated 12-well plate for further incubation. A one-third portion of fresh NB medium without glutamate was supplemented every 4 days to maintain cell health. Cell lines, including HEK293T (ATCC, CRL32160), HeLa cells (ATCC, CCL-2), and COS7 cells (ATCC, CRL-1651), were cultured in DMEM supplemented with 1× antibiotic–antimycotic, 1 mM sodium pyruvate, and 10% fetal bovine serum (FBS), which have been examined without mycoplasma.

For primary NSC culture, embryos at embryonic day 13.5 (E13.5) were used. The dorsal-telencephalon was dissected from the embryo, ensuring the removal of meninges. The dissected tissue was washed five times with HBSS without calcium and magnesium. Next, the buffer was replaced with 0.1 mL of papain solution per brain and incubated at 37 °C for 20 min for dissociation of tissue. After removing the papain solution, 1 mL of progenitor medium (consisting of DMEM/F12 medium supplemented with 2% B27 supplement, 0.2 mM glutamine, 2 μg/mL heparin, 10 ng/mL basic fibroblast growth factor, 20 ng/mL epidermal growth factor, and 1× antibiotic–antimycotic) was added to the tissue. The tissue was gently pipetted up and down 10 times using a 1000 μL tip, followed by centrifugation at 4 °C and 1000 rpm for 5 min. Finally, the cell pellet was resuspended in progenitor medium, and dissociated cells were seeded in a 12-well plate with coverslips. After 1 day of incubation, 50% of the medium was replaced with fresh medium for culture cell maintenance.

### Immunocytochemistry^72^

Cells cultured on PLL-coated coverslips were washed with warm PBS and then fixed with warm 4% PFA/PBS at 37 °C for 20 min. Permeablization was performed with 0.2% TX-100 in PBS at 4 °C for 30 min. Cells were then conducted for blocking with 5% BSA and 5% FBS in PBS for 1 h at RT. For immunofluorescence labeling, cells were treated with primary antibodies diluted in PBS containing 1% BSA, 1% FBS, and 0.05% Triton X-100. Following overnight incubations at 4 °C, cells were washed extensively with PBS containing 0.05% Triton X-100 and then incubated with secondary antibodies conjugated with Alexa fluorophores and DAPI. After PBS washing, coverslips were mounted in Prolong Gold mounting medium with antifade reagent (Invitrogen) for image acquisition. GFP and mCherry signals had no antibody enhancement.

### Microscopy and Image Acquisition^71,72^

For detecting GFP and mCherry positive cells, immunostained cells were observed with a confocal laser scanning microscope LSM780 (CarlZeiss, Germany) equipped with an argon laser (excitation 488 nm) and a DPS laser (excitation 561 nm). Serial optical *Z*-sections were acquired using a Plan Apochromat 20*X*/0.8 M27 objective with 2048 × 2048 resolution. The *x*–*y* plane resolution was calculated by scaling 0.231 μm with apertures of 27 μm. The neuronal architectures were detected by serial optical *Z*-sections using a Plan Apochromat 40*X*/1.4 Oil DIC M27 objective with a 2048 × 2048 resolution. The *x*–*y* plane resolution as calculated by scaling 0.148 μm with apertures of 29 μm. For live-image of synaptic plasticity, neurons cultured on PLL-coated coverslip were detected by serial optical *Z*-sections using a Plan Apochromat 63*X*/1.4 Oil DIC M27 objective with 2048 × 2048 resolution and 0.15% laser power at DIV25 or 65. The *x*–*y* plane resolution was calculated by scaling 0.066 μm with apertures of 80 μm. The interval time was 2 min for each image, and a recording was conducted for 10 h. Projection images were generated using ZEN-black image analysis software (Carl Zeiss, 2011 SP7 FP3, version 14.0.0.0) with full resolution and are shown for the architecture of neurons. For detecting CaP nanoparticle size, number, and movement, an Axiovert 40 CFL microscope equipped with an Axiocam 208 color monitor and Labscope APP (Carl Zeiss) was applied. The different power field images were acquired by LD A-Plan 20*X*/0.30 Ph (1006–591), LD A-Plan 40*X*/0.50 Ph2 (1006–595), Plan-POCHROMAT 63*X*/1.4 Oil DIC (420782–9900), and Plan-POCHROMAT 100*X*/1.4 Oil DIC (420792–9900) objectives, respectively.

### Image Analysis and Quantification^71^

All images were acquired with a fixed exposure time and conditions in the same experiment. All quantification was performed under raw 8- or 16-bit images. For CaP nanoparticle size and number, over 1000 particles were analyzed for each group from six independent experiments by threshold change and particle analysis plugins of ImageJ (Version1.53t). To address the population of GFP or mCherry positive cells, the cell counter plugin of ImageJ was applied, which was normalized by MAP2 positive cells to identify the real numbers of neurons. Moreover, the cell counter plugin of ImageJ was also applied to analyze the number of spines, dendrites, and dendritic branches in neurons. To verify the CMTR1 and CPEB2 protein intensity in shRNA knockdown (mCherry positive) and control (GFP positive) neurons, the Multi Measure plugin of ImageJ was conducted to quantify the specific protein intensity with the background correction within a cell. The protein transfection efficiency and degradation rate after teleofection were quantified by the intensity of a fluorescent protein signal with the Multi Measure plugin of ImageJ. All statistical analysis was performed by GraphPad Prism 9.0 and Microsoft Excel (see statistical analysis for the details).

### Cell Viability Assay

Rat neurons were conducted for cell viability assay at DIV10 and were divided into four groups including control, teleofection, Lipo-3000, and conventional CaP methods. For the transfection time, teleofection and conventional CaP methods were applied for 60 min; for Lipo-3000, neurons were consistently incubated in the commercialized reagent according to the user manual. 1× PrestoBlue Reagent, a ready to use cell-permeable resazurin-based colorimetric fluorescence solution, was obtained by mixing 10× Stokes with HBSS and introduced to measure cell viability at indicated time points. Before adding 1× PrestoBlue Reagent into culture neurons, it is critical to transfer and keep the conditioned culture medium for further incubation after analysis of each time point. An amount of 1 mL of 1× PrestoBlue Reagent was added into neurons and incubated at 37 °C and 5% CO_2_ incubator for 20 min, which was then recovered for spectrofluorometer analysis by a SpectraMax Gemini EM Microplate Reader (Molecular Devices) with emission of 590–615 nm and excitation of 535–560 nm.

### *In Vitro* Transcription

To obtain mRNA for teleofection, the ultrahigh yield mMESSAGE mMACHINE T7 *in vitro* transcription kit (ThermoFisher, AM1344) was applied. All reagents and linearized pT7-EGFP/N plasmid were mixed well and then incubated at 37 °C for 60 min for *in vitro* transcription according to the user manual. After digesting the plasmid by adding 1 mL of TURBO DNase (ThermoFisher, AM2238) and incubating for 15 min at 37 °C, the mixture was cleaned up by DyeExTM 2.0 Spin Columns 50 (QIAGEN, 63204) to remove free nucleotides, followed by phenol/chloroform extraction and ethanol precipitation. The quantity and quality of synthesized RNA were analyzed by NanoDrop Spectrophotometers (ThermoFisher, ND-2000) and agarose-gel electrophoresis analysis.

### Reporter Assay^38^

Primary neurons and stem cells with a density of 4 × 10^5^ cells were cultured in a 12-well plate. Teleofection was performed at DIV10 to deliver 1 μg of Firefly (*Photinus pyralis*) luciferase reporter appended with or without PDGFRα 3′-UTR and 0.1 μg of Renilla (*Renilla reniformis*) luciferase construct as an internal control. The neurons were harvested 24 h after teleofection for dual-luciferase assay and immunoblotting. The Dual-Luciferase Reporter Assay System (Promega) was applied to detect reporter gene activity. Before a lysis step, neurons were briefly washed with HBSS to remove detached cells and residual growth medium. Neurons were then treated with 250 μL of Passive Lysis Buffer (PLB) per well to promote the rapid lysis of neurons without the need to scrape or perform additional freeze–thaw cycles (active lysis). An amount of 20 μL of cell lysate was transferred into each luminometer tube containing 100 μL of Luciferase Assay Reagent II and mixed by pipetting 5 times for further luminescence intensity analysis. A Sirius Single Tube Luminometer was applied to detect luciferase activity by following the user manual. For the rest of the cell lysates, 5× protein loading dye was applied and mixed well with an equal amount of protein for Western blot assay.

### Western Blot Assay^72^

Protein extracts were prepared from equal amounts of the cultured neurons in Laemmli sample buffer supplemented with 1× protease inhibitor cocktail (Roche) and phosphatase inhibitors (Tocris). Homogenates were sonicated by an ultrasonic cell disruptor (Misonix Sonicator 3000) with power level 2 to disrupt genomic DNA, and insoluble debris were removed by centrifugation at 14000*g* and 4 °C for 10 min. Protein lysates were mixed well with 5× protein loading dye (312 mM Tris-HCl, pH6.8; 10% SDS; 45% Glycerol; 1% Bromophenol Blue; 5% β-mercaptoethanol) and boiled at 37 °C for 10 min, then resolved by 10 or 12% SDS-PAGE minigels, and then transferred to nitrocellulose membranes. The membranes were then blocked in 5% skim milk in 1× TBST (TBS with 0.1% Tween 20) for 60 min, followed by the incubation of the indicated primary antibodies at 4 °C overnight. Specific horseradish peroxidase-conjugated secondary antibodies were used for enhanced chemiluminescence detection (ECL-Prime, GE Healthcare Life Sciences) by an Image Quant LAS 4000 (Fujifilm).

### Whole-Cell Electrophysiology

Voltage-gated sodium current recordings were conducted at room temperature (25 ± 1 °C) in rat primary neuronal cells. We used a digidata 1440a digitizer, axopatch 200B amplifier, and Clampex program to record sodium current activity. The glass micropipettes would be fire-polished using a PP-830 puller. We implemented 80–90% series resistance compensation to reduce voltage errors and filtered with a low-pass Bessel setting of 5 kHz. The extracellular bath solution contained (in mM): 110 choline chloride, 20 NaCl, 2.5 KCl, 7 MgCl_2_, 0.5 CaCl_2_, 1.25 NaH_2_PO_4_·H2O, 25 NaHCO_3_, 11 glucose, 20 tetraethylammonium chloride (TEA·Cl), pH 7.30 with NaOH. The pipet solution for voltage-clamp recordings contained (in mM): 150 CsCl, 4 NaCl, 1 MgCl_2_, 10 HEPES, 0.5 EGTA, 5 Mg-ATP, 1 Na_2_GTP, 10 Creatine, adjusted to pH 7.3 with CsOH, under an osmolarity of 295–300 mOsm. Current–voltage (*I*–*V*) relationship was determined using 100 ms step depolarizations (−80 to +50 mV) in 10 mV increments (5 s intervals) from the holding potential.

### Fura-2-Dependent Calcium Image

Primary neurons cultured on coverslips were transferred from culture medium to HBSS containing 1 mg/mL BSA and 2 μM Fura-2 for dye loading at DIV14. Cells were incubated in a CO_2_/37 °C incubator for the 45 min loading time followed by HBSS wash to remove overloading dye three times. For live-image recording, Zeiss axio observer d1 with a 20*x* objective lens (Zeiss Plan Apochromatic 20*x*/0,60 Objective 440640) was conducted under a perfusion buffer system. The recording frequency was one frame per 1.5 s for 340/380 nm excitation and 510 nm emission signal acquisition. During image recording, cells were preincubated in teleofection medium without CaP nanoparticles for 10 min equilibrium, followed by 25 min CaP nanoparticle treatment. HBSS was applied to washout CaP nanoparticles for 5 min, following 50 μM NMDA/HBSS stimulation to induce extracellular Ca^2+^ influx for neuronal activity examination.

### Flow Cytometry

293T cells cultured in a 6-well plate were conducted to teleofection for 60 min with or without CaP nanoparticles, following 3 and 24 h incubation after particle washout by HBSS. Cell resuspension was performed by 1 mL 0.025% trypsin/PBS at 37 °C 5% CO_2_ incubator, for 2 min. Resuspended cells were fixed by 4% PFA/PBS at 4 °C overnight with gentle rotation, followed by PBS wash and 0.2% TX-100/PBS containing 0.25 mg/mL RNase A for cell permeabilization at 4 °C, for 30 min. The permeablized cells were stained with 10 μg/mL DAPI at room temperature for 60 min. The DNA content of labeled cells was measured by a flow cytometer (Becton Dickinson, LSRII SORP–17 color analyzer). Acquired data were analyzed by BD FACSDiva software v6.2.

### Recombinant Protein Purification^43,73^

GFP-CTTNBP2 and NuMA C-terminal tail domain-GFPs (a.a. 1868–2091; NuMA tail II) were expressed in insect cell and bacterial systems and purified by chromatography according to the published protocol (PMID: 35562389; PMID: 28939615). In brief, we used the Multibac system to generate baculovirus strains expressing GFP-tagged full-length CTTNBP2. Protein was purified by an lgG-Sepharose 6 fast flow affinity column (GE Healthcare) and a 10/300 Superdex 200 column. NuMA tail II was expressed in the bacterial strain Rosetta (Novagen). Subsequently, the protein was purified by Ni resin (Sigma), followed by Hi-Trap Q HP and Superdex 200 (16/60) columns. Ultimately, both purified proteins were concentrated and stored at −80 °C.

### Statistical Analysis

Cultured neurons and cell lines were randomly assigned for all the experiments. Imaging fields were randomly selected during image acquisition. The sample size among experimental groups was kept as equally as possible. The experiments and analysis were conducted in a blind manner and replicated at least three times independently. GraphPad Prism 9.0 software was applied to produce the graphs and statistical analysis. Sample numbers and statistical results were indicated in the figure legends precisely. Data were presented as the mean ± standard error (s.e.m.), and *P* values less than 0.05 were considered statistically significant. *P* values are represented as *P < 0.05, **P < 0.01, ***P < 0.001, and ****P < 0.0001.

We conducted experiments including CMTR1 intensity (Figure 3H), dendrite number (Figure 3I), dendrite length (Figure 3J), dendrite branch (Figure 3K), luciferase activity (Figures 4H and 6E), the transfection efficiency between the rat and mouse (Supporting Information Figure S4E), CPEB2 intensity (Supporting Information Figure S7G), spine density (Supporting Information Figure S7I), protein degradation rate (Supporting Information Figure S9E), and effect of CaP nanoparticles on cell division (Supporting Information Figure S9H) to a two-tailed Mann–Whitney nonparametric unpaired Student’s *t* test. For the effect of calcium concentration, media and buffers on nanoparticle size and number (Figure 1B, 1C, 1E, 1G, 1H, 1J, 1L, and 1M), time, and dose effects on teleofection efficiency (Figure 2B, 2D, 6B and Supporting Information Figures S5B, S5C, and S9B), the numbers of GFP and MAP2 positive cells (Figure 1E and J and Supporting Information Figure S2C), the effect of endocytosis and importin inhibitors (Supporting Information Figure S3B and S3C), calcium image (Supporting Information Figure S3E), apoptosis state (Supporting Information Figure S4D), transfection efficiency of commercial kits (Supporting Information Figure S4F and S4I), the population of Nestin and Notch1 double-positive cells (Supporting Information Figure S9A), protein delivery efficiency, and degradation rate (Figures 5D,E and 6F and Supporting Information Figure S8F,G) were statistically analyzed by one-way ANOVA with Bonferroni post-test. For the comparison of GFP-positive neuron and glia (Figure 2F and 2G), comparison of spiny and aspiny neurons (Figure 2H), and cell viability after transfection (Supporting Information Figures S2E,F and S4B) were analyzed by Two-way ANOVA with Bonferroni post-test.

## Supporting Information Available

Figure S1: The flowchart of the sequential steps for teleofection. Figure S2: The size and number of CaP nanoparticles are collectively influenced by calcium concentrations, medium types, and buffer systems. Figure S3: Characterizing the impact of CaP nanoparticles on cellular physiology and their delivery efficacy in primary neurons. Figure S4: Comparison of teleofection with currently popular methods under different states. Figure S5: The characterization of teleofection in cell lines. Figure S6: Neuronal architecture could be outlined precisely by teleofection. Figure S7: Teleofection enables the examination of various gene manipulations within the same culture. Figure S8. Teleofection enables the delivery of functional proteins into primary neurons. Figure S9. Characterizing the capabilities and impacts of teleofection in NSCs. Table S1: Comparison of teleofection to the most common used DNA transfection methods. Table S2: Summary of the main synthesis methods for CaP nanoparticles. Table S3: Summary of conventional methods for CaP particle generation. Table S4: The volumes and the most common sizes for the transfection experiment. Table S5: The plasmid information. Table S6: Current methods for RNA transfection in neurons and NSCs (PDF)
CaP nanoparticles generated by teleofection. The CaP nanoparticles with ∼100 nm diameter are small enough to perform extensive and strong Brownian motions (AVI)
CaP particles generated by a conventional method. The CaP particles show micrometer scale in size and perform very weak Brownian motions (AVI)
Liposomes generated by Lipofectamin 3000 display nanoscale size (AVI)
Video displaying the mobility of a GFP-outlined stubby spine after teleofection (AVI)
Video displaying the mobility of a GFP-outlined branched spine after teleofection (AVI)
Video displaying the mobility of a GFP-outlined thin spine after teleofection (AVI)
Video displaying the mobility of a GFP-outlined axon terminal after teleofection (AVI)
Video displaying the mobility of a GFP-outlined En passant boutons axon after teleofection (AVI)
Video displaying the mobility of a GFP-outlined terminaux boutons axon after teleofection (AVI)
Dynamic interaction of the spine and axon (type 1). Serial teleofection makes axons (red) and dendritic spines (green) from heterotypic neurons to be distinguished using different colors, allowing for real-time monitoring of pre- and post-synaptic interactions. The video is related to Figure 4d in the main text (AVI)
Dynamic interaction of the spine and axon (type 2). Serial teleofection makes axons (red) and dendritic spines (green) from heterotypic neurons to be distinguished using different colors, allowing for real-time monitoring of pre- and post-synaptic interactions. The video is related to Figure 4d in the main text (AVI)
Dynamic interaction of the spine and axon (type 3). Serial teleofection makes axons (red) and dendritic spines (green) from heterotypic neurons to be distinguished using different colors, allowing for real-time monitoring of pre- and post-synaptic interactions. The video is related to Figure 4d in the main text (AVI)

## Supplementary Material

nn3c09608_si_001.pdf

nn3c09608_si_002.avi

nn3c09608_si_003.avi

nn3c09608_si_004.avi

nn3c09608_si_005.avi

nn3c09608_si_006.avi

nn3c09608_si_007.avi

nn3c09608_si_008.avi

nn3c09608_si_009.avi

nn3c09608_si_010.avi

nn3c09608_si_011.avi

nn3c09608_si_012.avi

nn3c09608_si_013.avi
