# Supplementary material for: Improved CaP Nanoparticles for Nucleic Acid and Protein Delivery to Neural Primary Cultures and Stem Cells
Source: ACS Nano. 2024 Jan 29;18(6):4822–39. doi: 10.1021/acsnano.3c09608 (PMC10867895; doi:10.1021/acsnano.3c09608)
Supplement: Supplementary file 1 — nn3c09608_si_001.pdf [file nn3c09608_si_001.pdf]

Supporting Information

TITLE

Improved CaP Nanoparticles for Nucleic Acid and Protein Delivery to Neural Primary Cultures and Stem Cells

AUTHORS

Yu-Wen Chao[1,2,¥], Yen-Lurk Lee[3,4,¥], Ching-San Tseng[5,¥], Lily Ueh-Hsi Wang[4,#], Kuo-Chiang Hsia[4,#], Huatao Chen[6,7], Jean-Michel Fustin[8], Sayma Azeem[3,9], Tzu-Tung Chang[3], Chiung-Ya Chen[4], Fan-Che Kung[3], Yi-Ping Hsueh[4,⋆], Yi-Shuian Huang[3,9,10⋆], Hsu-Wen Chao[1,2,11,¥,⋆]

AFFILIATIONS

[1] Department of Physiology, School of Medicine, College of Medicine, Taipei Medical University, Taipei 110301, Taiwan

[2] Graduate Institute of Medical Sciences, College of Medicine, Taipei Medical University, Taipei 110301, Taiwan

[3] Institute of Biomedical Sciences, Academia Sinica, Taipei 115201, Taiwan.

[4] Institute of Molecular Biology, Academia Sinica, Taipei 115201, Taiwan

[5] Department of Anatomy, School of Medicine, China Medical University, Taichung 40402, Taiwan

[6] Department of Clinical Veterinary Medicine, College of Veterinary Medicine, Northwest A&F University, Yangling, Shaanxi 712100, China

[7] Key Laboratory of Animal Biotechnology of the Ministry of Agriculture and Rural Affairs, Northwest A&F University, Yangling, Shaanxi 712100, China

[8] The University of Manchester, Faculty of Biology, Medicine and Health, Oxford Road, Manchester M13 9PL, UK

[9] Taiwan International Graduate Program in Interdisciplinary Neuroscience, National Yang-Ming Chao-Tung University and Academia Sinica, Taipei 115201, Taiwan

[10] Institute of Molecular Medicine, College of Medicine, National Taiwan University, Taipei 10002, Taiwan

[11] Department of Biomedical Science and Environmental Biology, Kaohsiung Medical University, Kaohsiung, 80708, Taiwan

∗ CORRESPONDENCE:

1. Hsu-Wen Chao,

Taipei Medical University, No. 250, Wu-Hsing Street, Taipei city, 11031, Taiwan. E-mail: chaohw3619@tmu.edu.tw; Phone: +886-2-2789-9133

2. Yi-Shuian Huang,

Institute of Biomedical Sciences, Academia Sinica, No. 128, Sec. 2, Academia Rd, Taipei, 11529, Taiwan. E-mail:yishuian@ibms.sinica.edu.tw; Phone: +886-2-2652-3523

3. Yi-Ping Hsueh,

Institute of Molecular Biology, Academia Sinica, No. 128, Sec. 2, Academia Rd, Taipei, 11529, Taiwan. E-mail:yph@gate.sinica.edu.tw; Phone: +886-2-2789-9311

CONTRIBUTIONS:

¥, #, These authors contributed equally to this work.

KEYWORDS:

Calcium phosphate, nanoparticles, transfection, gene delivery, primary neurons, neural stem cells.

TABLE of CONTENTS

Supplementary Figure S1....4

Supplementary Figure S2....6

Supplementary Figure S3....9

Supplementary Figure S4....12

Supplementary Figure S5....15

Supplementary Figure S6....17

Supplementary Figure S7....21

Supplementary Figure S8....23

Supplementary Figure S9....26

Supplementary Table S1....29

Supplementary Table S2....30

Supplementary Table S3....31

Supplementary Table S4....32

Supplementary Table S5....33

Supplementary Table S6....34

Supplementary References....35

Supplementary Figure S1

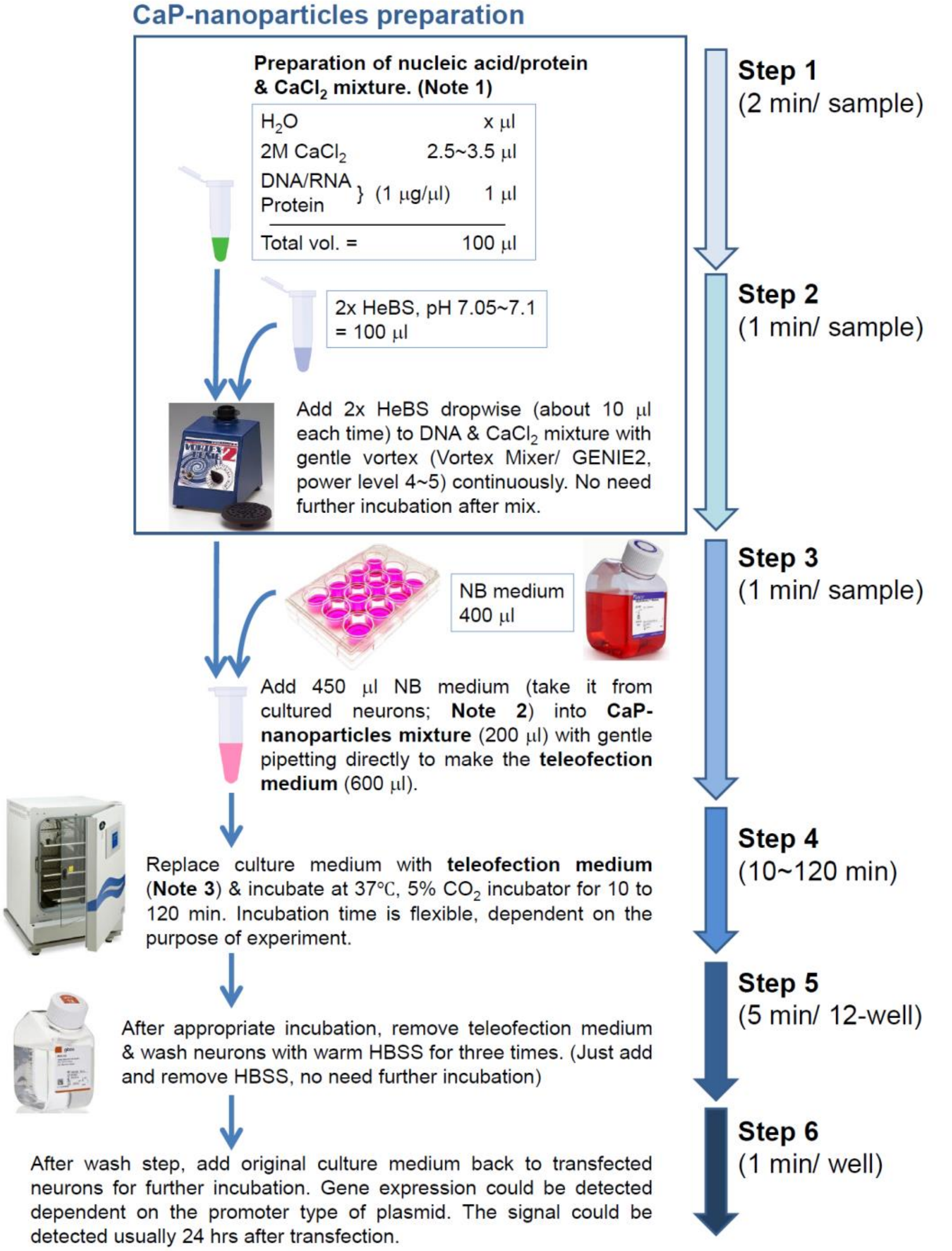

Note:

1. This protocol is the example for neurons cultured in 12-well plate; for the other culture conditions, adjust the volume based on the ratio of the surface of culture plate. (See **Table S4** and **Experimental Section** for the details)
2. Make sure your cultured neurons have enough conditioned medium for transfection. At least 1.5 ml/ well NB medium in your 12-well culture neurons is necessary.
3. For cell lines, the cell-specific culture media should be removed and replaced by NB-contained teleofection medium during transfection. (See **Experimental Section** for the details)

Supplementary Figure S1. The flowchart of the sequential steps for teleofection.

(**A**) Six simple steps of teleofection from preparing nucleic acid- or protein-CaP-nanoparticles to successful materials delivery. Step 1-2, CaP-nanoparticles preparation. To prepare DNA-, RNA-, or protein-CaP-nanoparticles for teleofection, 2x HeBS was added dropwise (about 10 μl each time) to the mixture with gentle vortex (Vortex Mixer/ GENIE2, power 4-5) continuously. No need further incubation after mixing. Step 3, add 450 μl Neurobasal (NB) medium into 200 μl teleofection mixture directly and pipetting it well to get transfection medium. Step 4, replace culture medium with transfection medium & incubate at 37℃, 5% CO_2 incubator from 10 min to 120 min to get the appropriate transfection efficiency. Incubation time is flexible, dependent on the purpose of experiments. Step 5, after appropriate incubation, remove transfection medium & wash cells with warm HBSS for three times. Just add and remove HBSS no need further incubation. Step 6, replace HBSS by conditioned NB culture medium. Gene expression could be detected dependent on the promoter type of plasmid. The signal could be detected usually 24~48 h after transfection.

Supplementary Figure S2

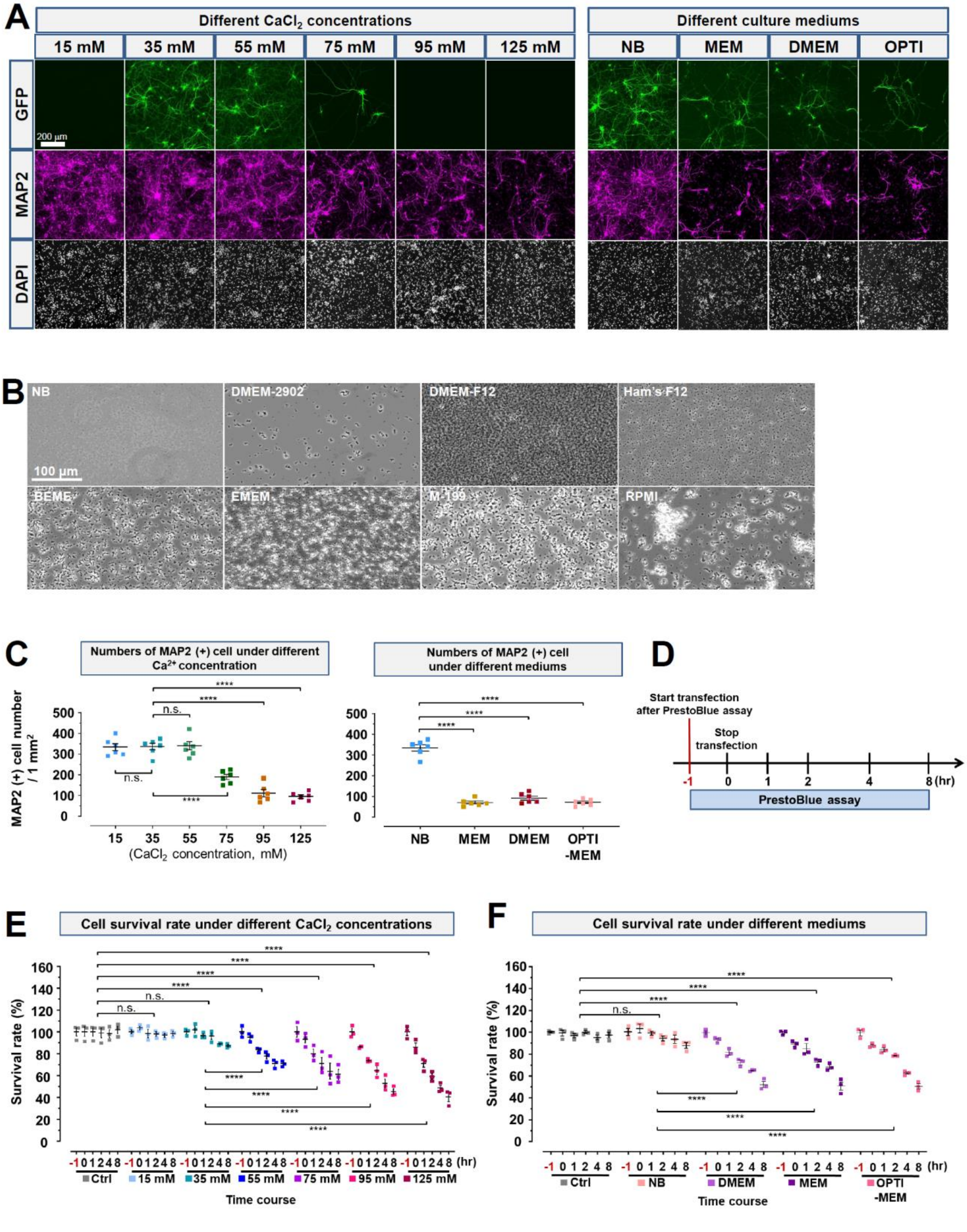

G

Item	2x HeBS Condition	pH	Nanoparticles formation	Transfection efficiency > 10%
1	Fresh preparation	7.04~7.06	Yes	Yes
2	-20℃ storage 1 year	7.04~7.06	Yes	Yes
3	21℃ storage 6 months	7.04~7.06	Yes	Yes
4	Freezing-and-thawing x 5	7.04~7.06	Yes	Yes
5	Heat-shock at 85℃	7.04~7.06	Yes	Yes

H

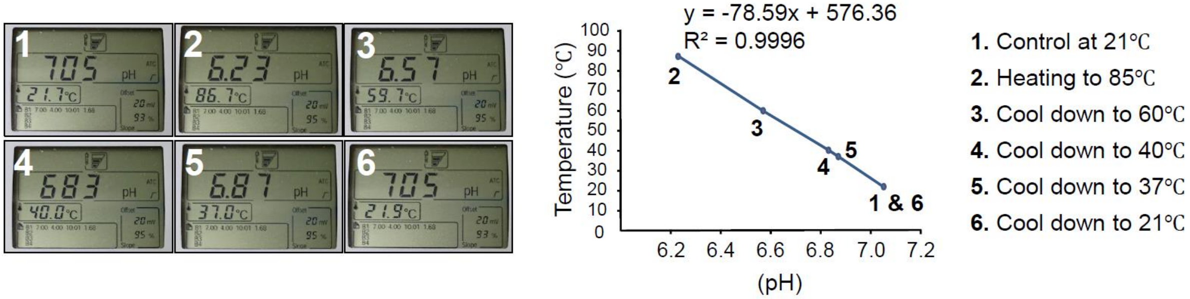

I

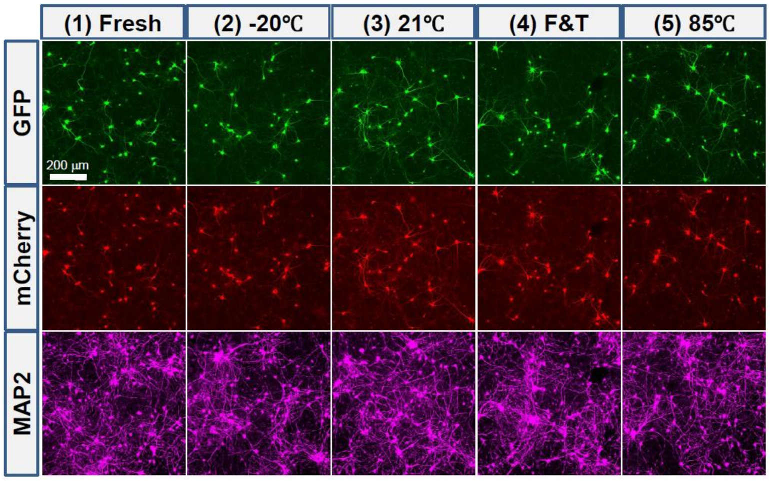

Supplementary Figure S2. The size and number of CaP-nanoparticles are collectively influenced by calcium concentrations, medium types, and buffer systems.

(**A**) The example images show the GFP- and MAP2-positive neurons after teleofection under indicated $CaCl_2$ concentrations and media. (**B**) The representative images manifest the size and morphology of CaP-particles in indicated culture media. (**C**) The quantitative data represents the numbers of MAP2-positive cell after teleofection under indicated $CaCl_2$ concentrations and media. (**D**) The flowchart of cell viability assay throughout DNA transfection. (**E**-**F**) The quantitative data indicate the cell viability throughout DNA transfection under indicated conditions. (**G**) The table illustrates the pH values, the capacity for CaP-nanoparticle formation, and transfection efficiency achieved through the utilization of indicated 2x HeBS systems. (**H**) The crop images of pH meter and line graph demonstrate the robust recovery and stability of 2x HeBS pH values under extreme temperature variations. (**I**) The representative images show the GFP and mCherry positive neurons after co-transfection via teleofection using the specified 2x HeBS systems. Statistic: One-way ANOVA, (**C**); Two-way ANOVA, (**E**-**F**). Values represent the mean ± s.e.m., *P < 0.05, **P < 0.01, ***P < 0.001, ****P < 0.0001. (These data are correlated to Figure 1).

Supplementary Figure S3

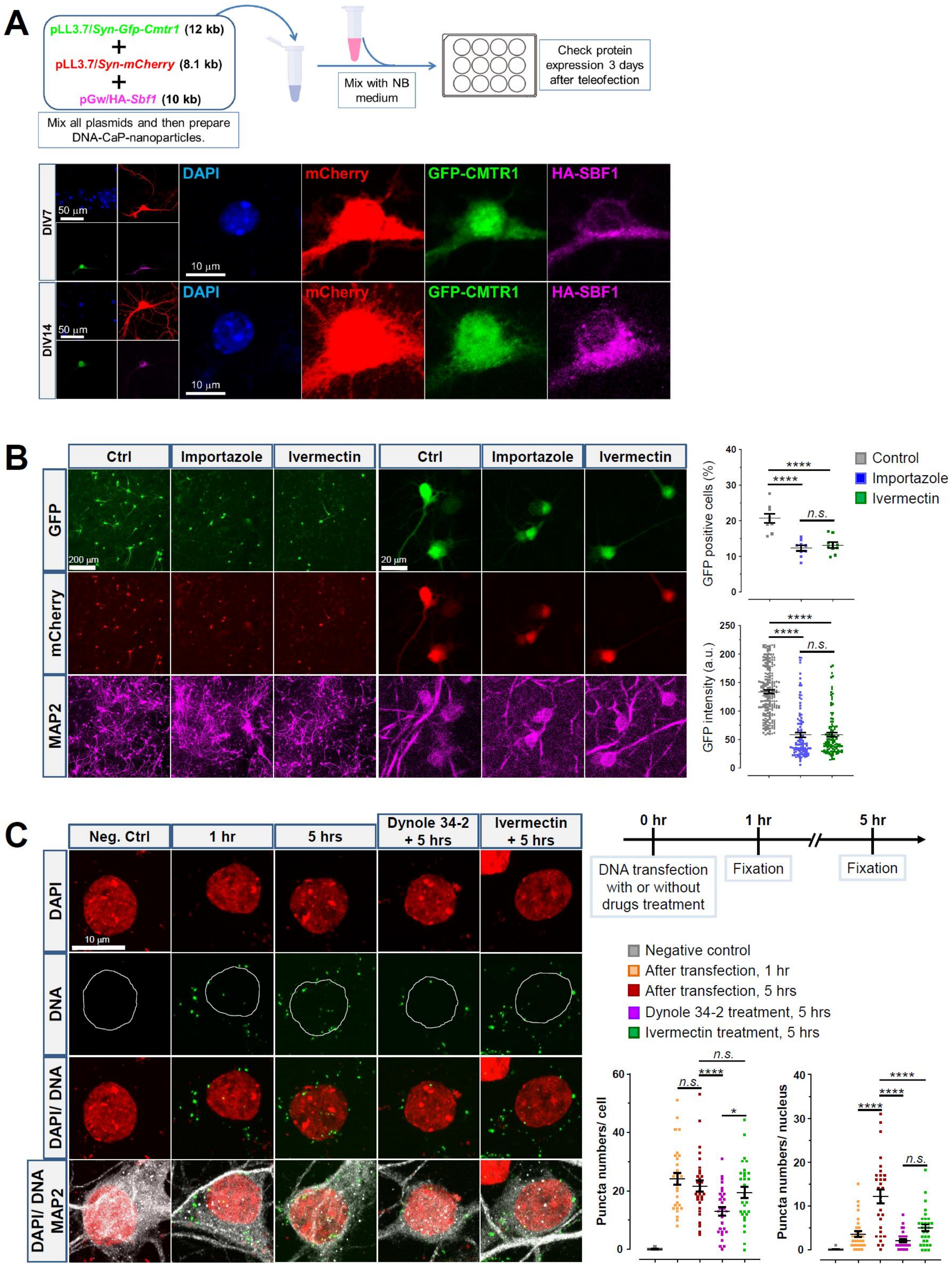

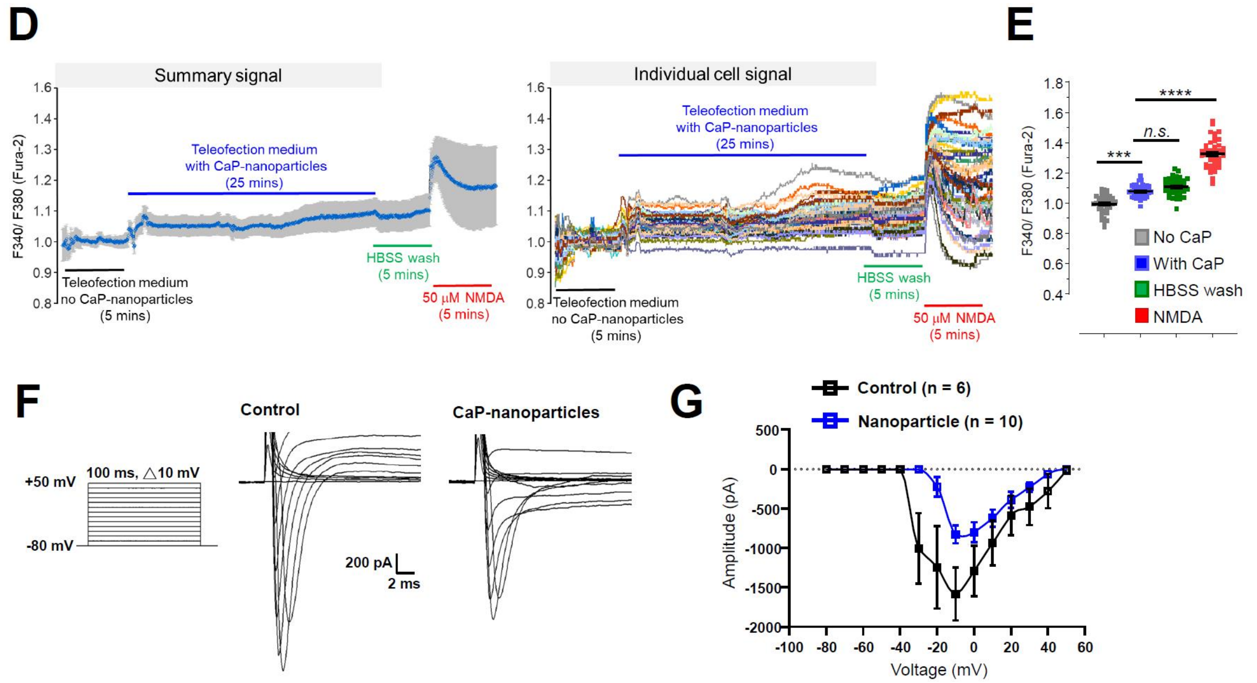

Supplementary Figure S3. Characterizing the impact of CaP-nanoparticles on cellular physiology and their delivery efficacy in primary neurons.

(**A**) The flowchart shows the experiment design for co-transfection of large molecule weight plasmids into primary neurons. Three kinds of plasmids were mixed together to generate DNA-CaP-nanoparticles, followed by combining with NB medium. The example images show the expression of mCherry (red), GFP-CMTR1 (green, nucleus distribution) and HA-SBF1 (magenta, cytosolic distribution) after teleofection in DIV7 and DIV14 primary neurons, indicating the capability of teleofection for co-transfection of large molecule weight nucleic acids. (**B**) The representative images show the effect of importazole (importin-β inhibitor) and ivermectin (importin-α/β1 inhibitor) on nucleus transportation and gene expression of transfected plasmids after teleofection. Primary neurons were co-transfected with pLL3.7/*Syn-Gfp* and *-mCherry* at DIV7, followed by importin inhibitors treatment (15 μM importazole and 2.5 μM ivermectin) for 5 h immediately and fixation at DIV10 for images acquisition. The quantitative data display that importin inhibitors reduce DNA transfection efficiency and GFP expression level. Over hundred MAP2-positive neurons from three different wells were analyzed. (**C**) The representative images illustrate the impact of endocytosis inhibitor (Dynole 34-2, 10 μM) and nuclear transporter inhibitor (ivermectin, 2.5 μM) on DNA delivery in primary

neurons. Teleofection was utilized to transfect a fluorescence (488)-conjugated plasmid with or without the application of the specified drugs, followed by fixation at the indicated time points. The dash-line circle demarcates the location and area of the nucleus. The dot plot graphs present quantitative data on the subcellular distribution of the transfected plasmid under the indicated conditions. Thirty neurons were analyzed from three independent coverslips. (**D**) The line graphs show the Fura-2-dependent intracellular change of calcium ($[Ca^{2+}]_{ic}$), displaying as F348/ F380 ratio. The average signal from fifty cells and the signal from individual cell were represent as left and right graphs respectively. (**E**) The dot plot graph indicates the $[Ca^{2+}]_{ic}$ under indicated conditions. Around 10% increase of $[Ca^{2+}]_{ic}$ was observed after CaP-nanoparticles administration. (**F**-**G**) The effect of CaP-nanoparticles on the electrophysiology of primary neurons was manifested as line graphs, displaying as sodium channel traces and I/V curves. Statistic: One-way ANOVA, (**B**, **C** and **E**). Values represent the mean ± s.e.m., **P* < 0.05, ***P* < 0.01, ****P* < 0.001, *****P* < 0.0001, n.s. = no statistic significance. (These data are correlated to Figure 1).

Supplementary Figure S4

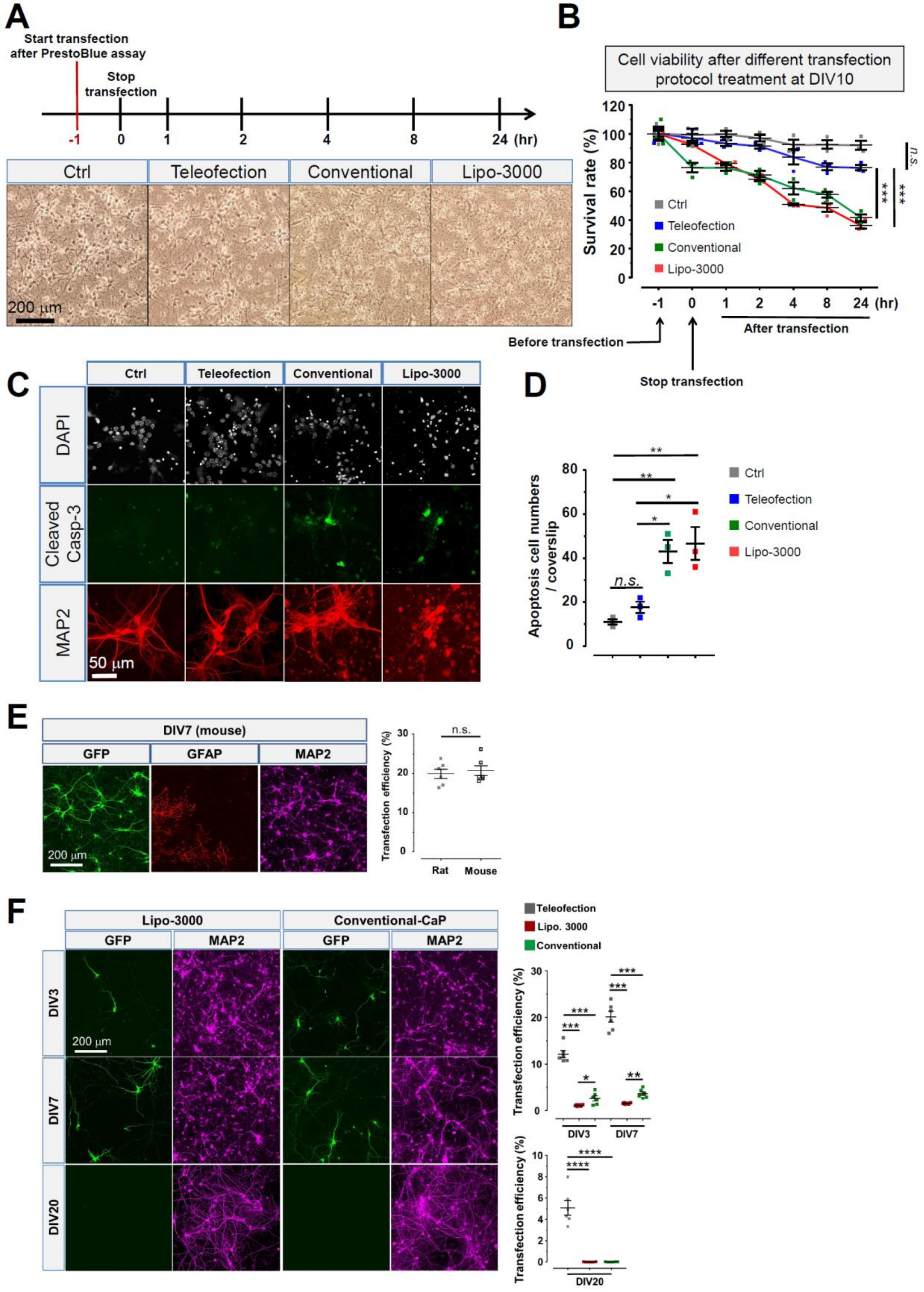

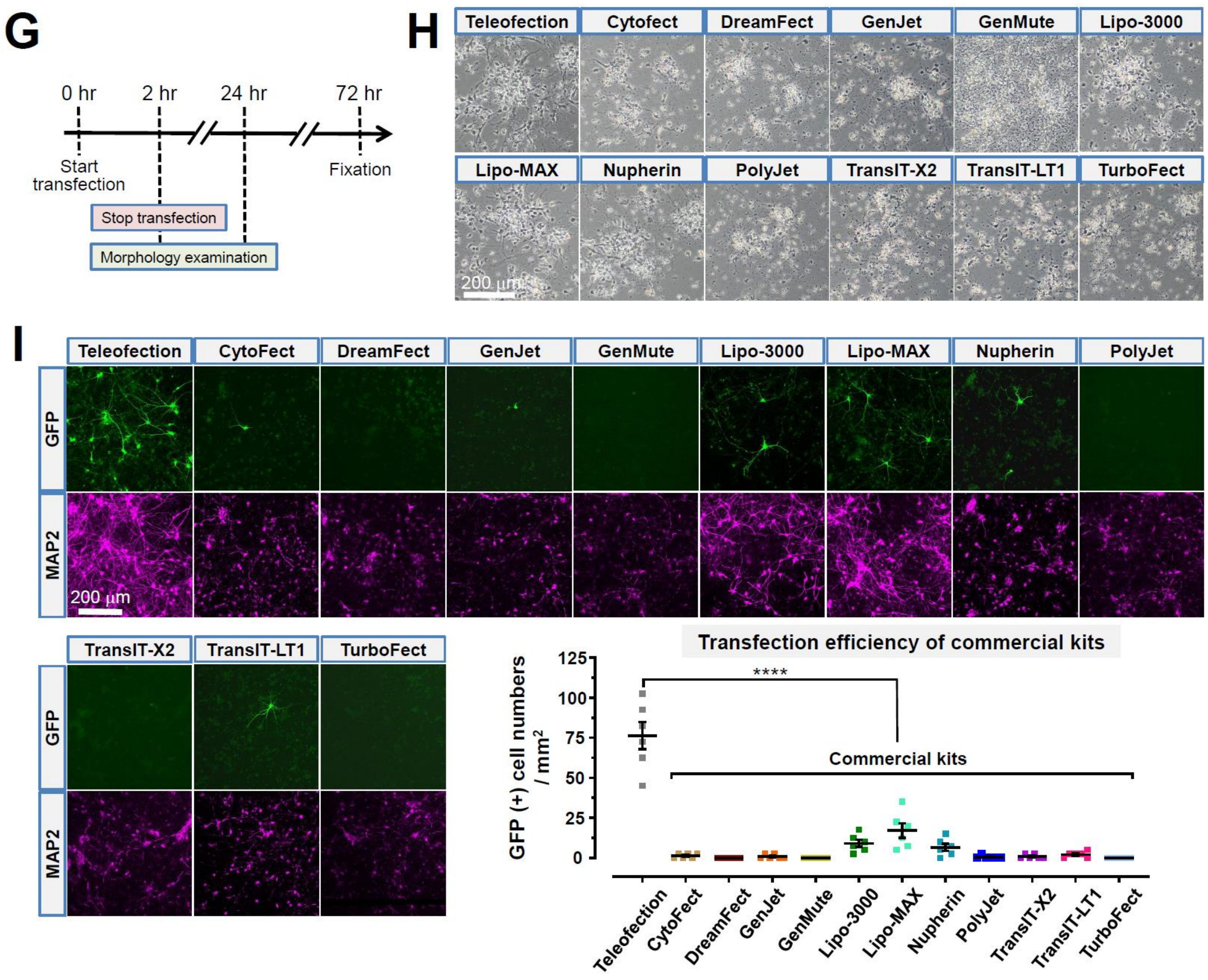

Supplementary Figure S4. Comparison of teleofection with currently popular methods under different states.

(**A**-**B**) Cytotoxicity was examined among control, teleofection, conventional CaP transfection, and Lipofectamine 3000 (Lipo-3000) groups, showing higher viability of teleofection at indicated time points. The bright field images display the morphology of primary neurons 24 h after transfection. (**C**-**D**) The representative images illustrate the apoptosis state following DNA transfection under the specified conditions. Primary neurons underwent various transfection protocols, followed by a 3 h incubation at DIV10. Cleaved Caspase-3 and MAP2 were probed using specific antibodies to monitor the apoptosis state and neuron morphology, respectively. The dot plot graph shows the quantitative data from three independent coverslips for each group. (**E**) The example images show that teleofection can be applied in mouse primary neuron culture, which

has similar transfection efficiency with rat primary neurons at DIV7. (**F**) The example images show the numbers of GFP positive neurons transfected by Lipo-3000 and conventional CaP at DIV3, 7 and 20. The quantitative data display the transfection efficiency in primary neurons at indicated times. Data were analyzed from three independent experiments with six different regions. (**G**-**H**) The flowchart illustrates the experiment design for transfection of pLL3.7/*Syn-Gfp* into primary neurons by indicated methods. The examples of images show the neural morphology after 2 h of transfection. (**I**) The representative images and quantitative data display the transfection efficiency of indicated methods. For the cell viability assay, data were analyzed from three independent experiments, while the remaining data were collected from six different areas across three independent experiments. Statistic: Two-way ANOVA, (**B**); Student's unpaired t-test (**C**); One-way ANOVA, (**D**, **F**, and **I**). Values represent the mean ± s.e.m., *$P < 0.05$, **$P < 0.01$, ***$P < 0.001$, ****$P < 0.0001$. (These data are correlated to Figure 2).

Supplementary Figure S5

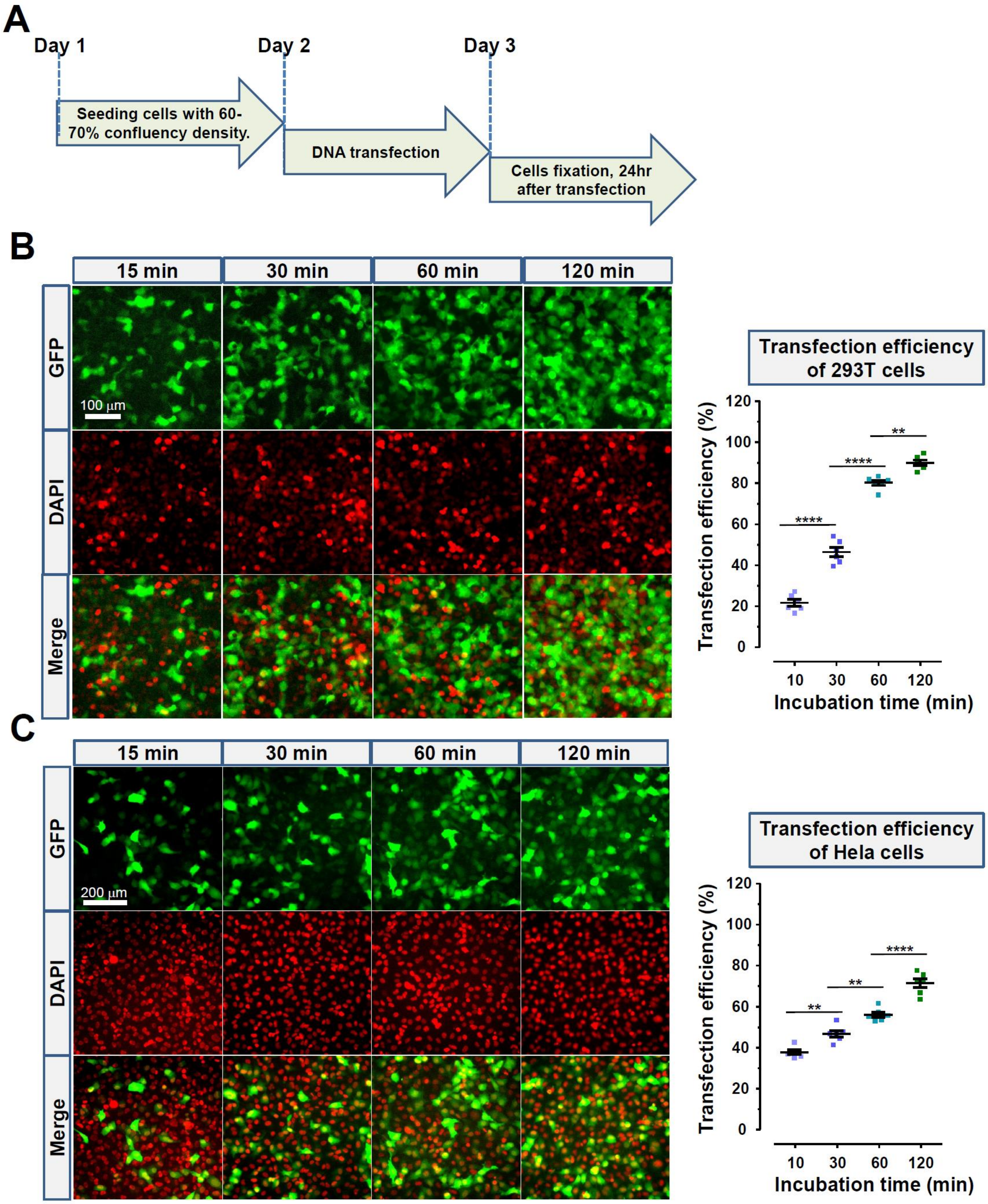

Supplementary Figure S5. The characterization of teleofection in cell lines.

(**A**) The flowchart shows the experiment design for transfection of pEGFP-C3 by teleofection. (**B**-**C**) The representative images show the expression level of GFP under time-dependent manners in transfected HEK293T and Hela cells. GFP was displayed as green, and nuclei were outlined by DAPI with red. The quantitative data were expressed as dot plot graphs that show significant increase of transfection efficiency dependent on the incubation time of transfection. Data were analyzed from six different areas across three independent experiments. Statistic: One-way ANOVA, (**B** and **C**). Values represent the mean ± s.e.m., **P* < 0.05, ***P* < 0.01, ****P* < 0.001, *****P* < 0.0001. (These data are correlated to Figure 2).

Supplementary Figure S6

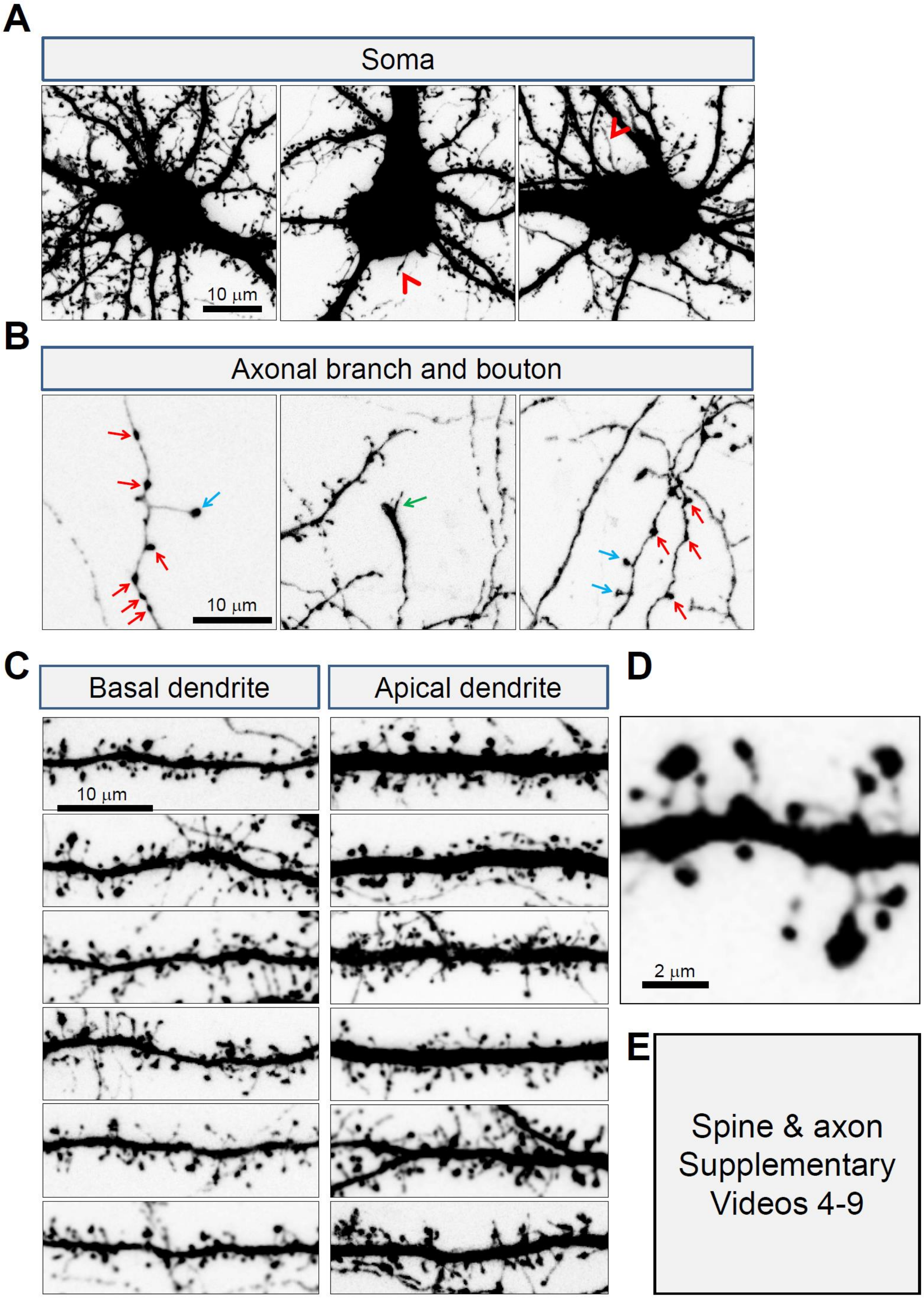

F

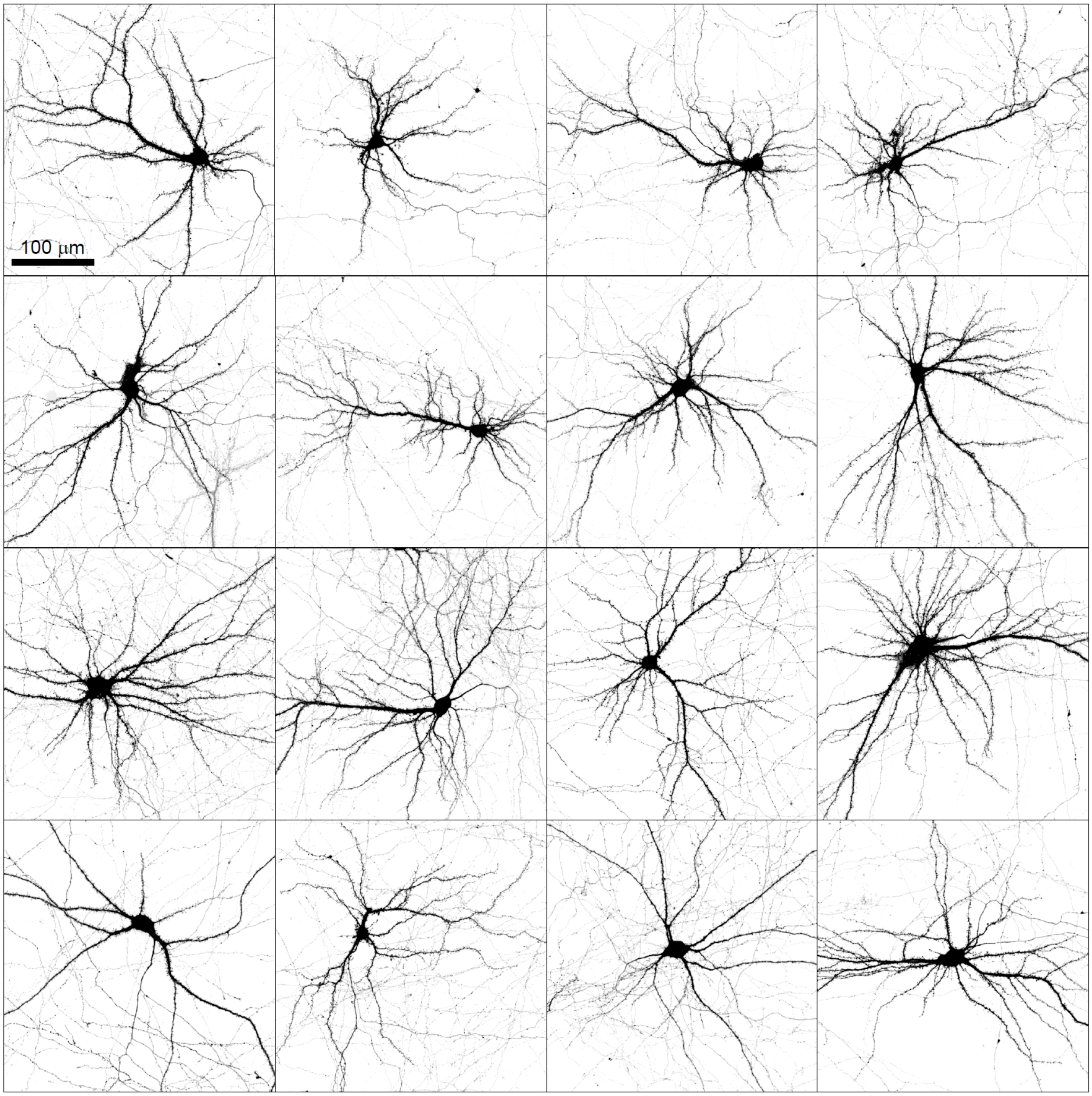

F (continued)

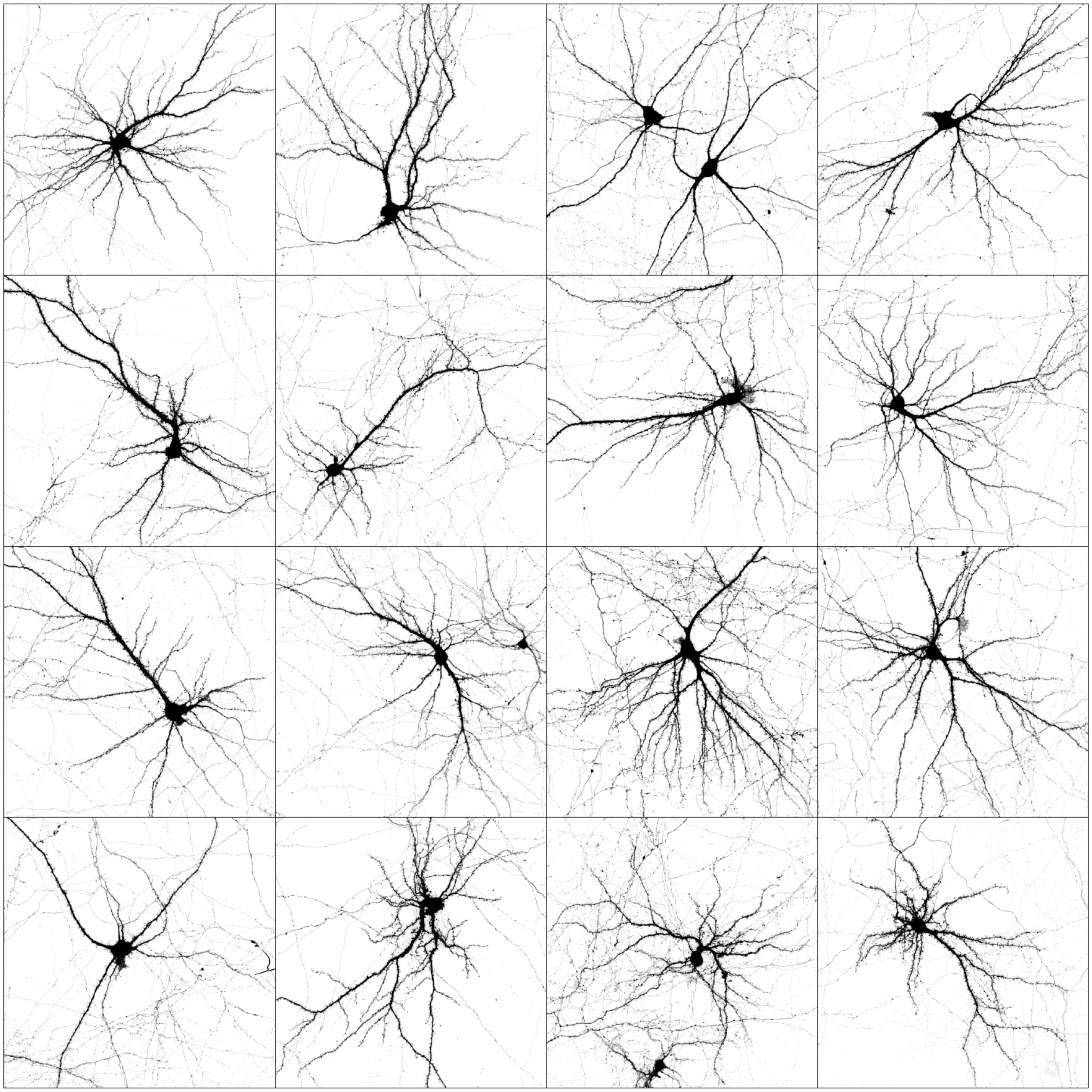

Supplementary Figure S6. Neuronal architecture could be outlined precisely by teleofection.

Neurons could at DIV30 still maintain health state and morphology after pLL3.7/*Syn-Gfp* transfection. The detail profile of soma (**A**), axon (**B**), and dendritic spine (**C**) could be obtained after teleofection. The examples of high magnification image of dendritic spines display as (**D**). Cilias (red arrow heads); terminaux boutons (blue arrows); en passant boutons (red arrows); growth cone (green arrows). (**E**) The Supplementary Videos S4-S9 show the dynamic movement of thin spine (Video S4), stubby spine (Video S5), branched spine (Video S6), axon terminal (Video S7), axon en passant boutons (Video S8), axon terminaux boutons (Video S9). The images were acquired with 0.05% laser power and 2 min interval for long-term recording. (**F**) Teleofection can deliver nucleic acid into various types of primary neurons. At least 32 different types of spiny neurons were identified with GFP positive signal. (These data are correlated to Figure 2 and 3)

Supplementary Figure S7

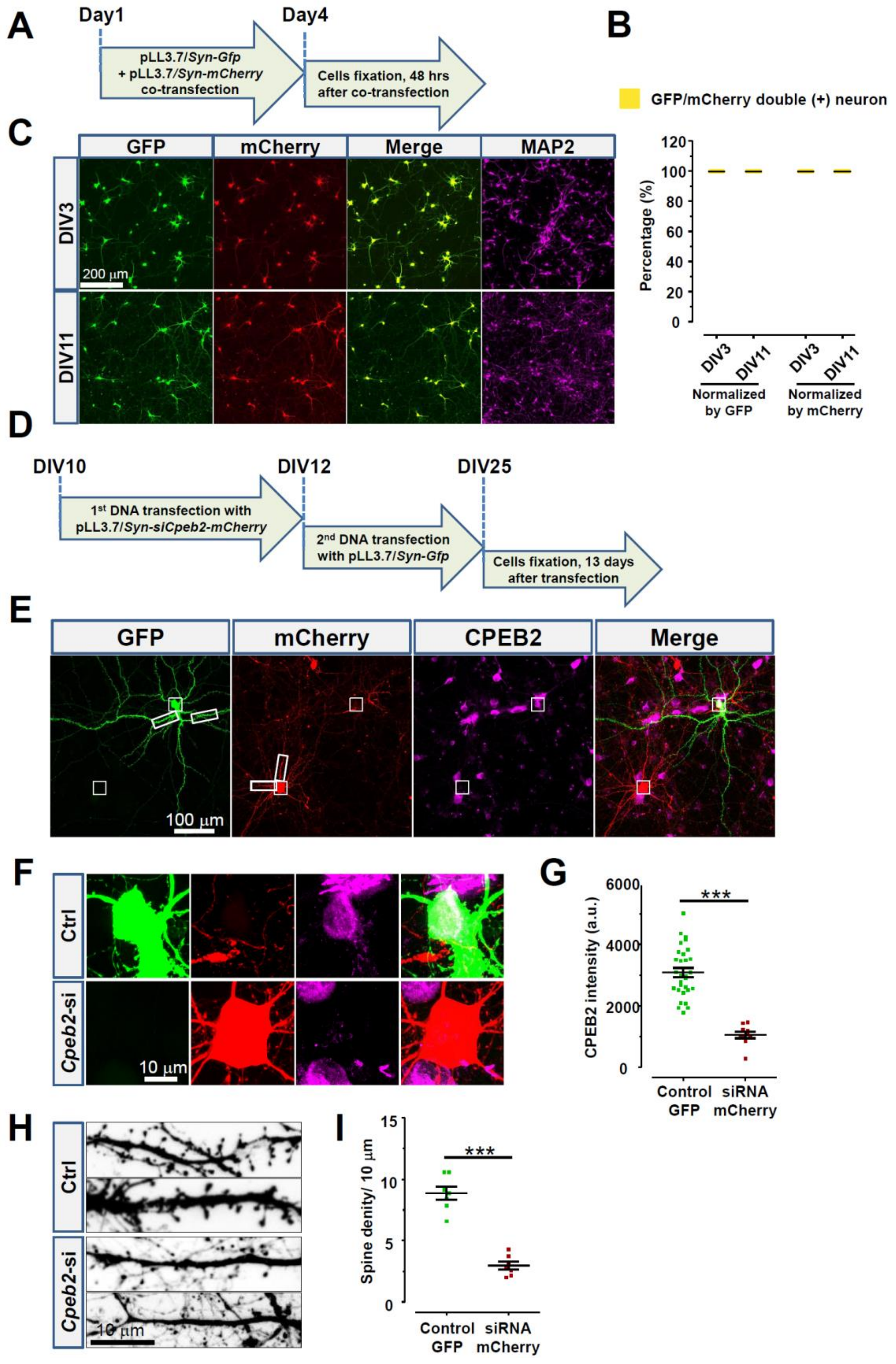

Supplementary Figure S7. Teleofection enables the examination of various gene manipulations within the same culture.

(**A**-**C**) The flowchart shows the experiment design for co-transfection of plasmids expressed GFP and mCherry. The illustrative images and quantitative data demonstrate that neurons expressing GFP (green) also exhibited mCherry positivity (red), indicating that simultaneous co-transfection of GFP and mCherry results in the co-expression of exogenous genes within the transfected neurons. Neurons were outlined by MAP2 signal in magenta. (**D**) The flowchart shows the experiment design for serial-transfection of *Cpeb2* siRNA, followed by pLL3.7/*Syn-Gpf* transfection at DIV7 and 10, respectively. The neurons were fixed for ICC at DIV20. (**E**) The representative images show the neurons expressed GFP (green) and mCherry (red) as control and *Cpeb2* knockdown groups, respectively. CPEB2 signal was displayed as magenta. (**F**) The high magnification images from **E** show the details of transfected neurons. (**G**) The quantitative data from **E** and **F** represents CPEB2 intensity in control and *Cpeb2* siRNA knockdown groups. (**H**) The high magnification images illustrate the details of dendritic spine in control and *Cpeb2* siRNA knockdown groups. (**I**) The quantitative data of **H** displays the density of dendritic spine from indicated groups. The data were analyzed from six different areas of three independent experiments. Statistic: Student's unpaired t-test (**G** and **I**). Values represent the mean ± s.e.m., *P < 0.05, **P < 0.01, ***P < 0.001, ****P < 0.0001. (These data are correlated to Figure 3).

Supplementary Figure S8

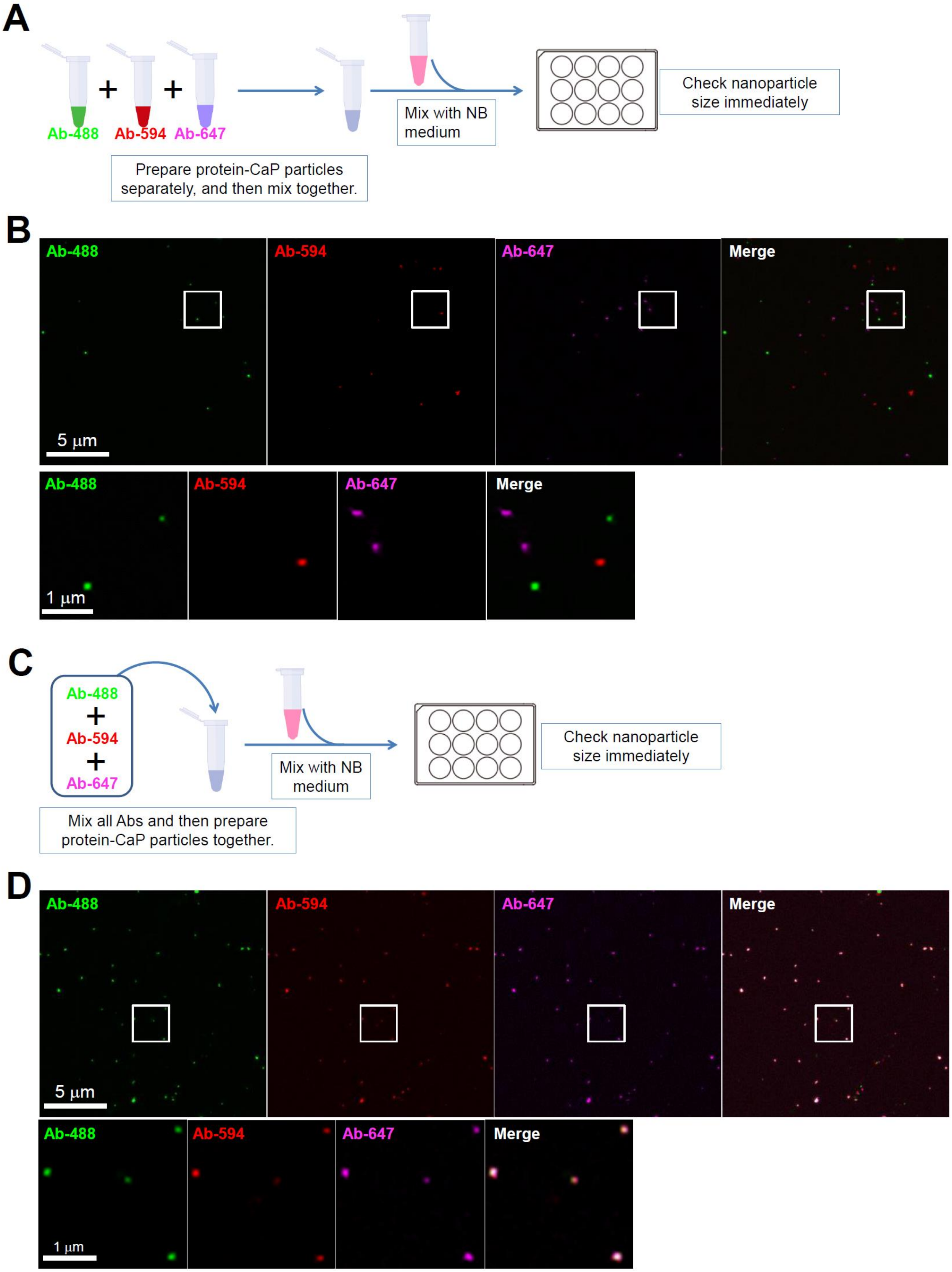

E

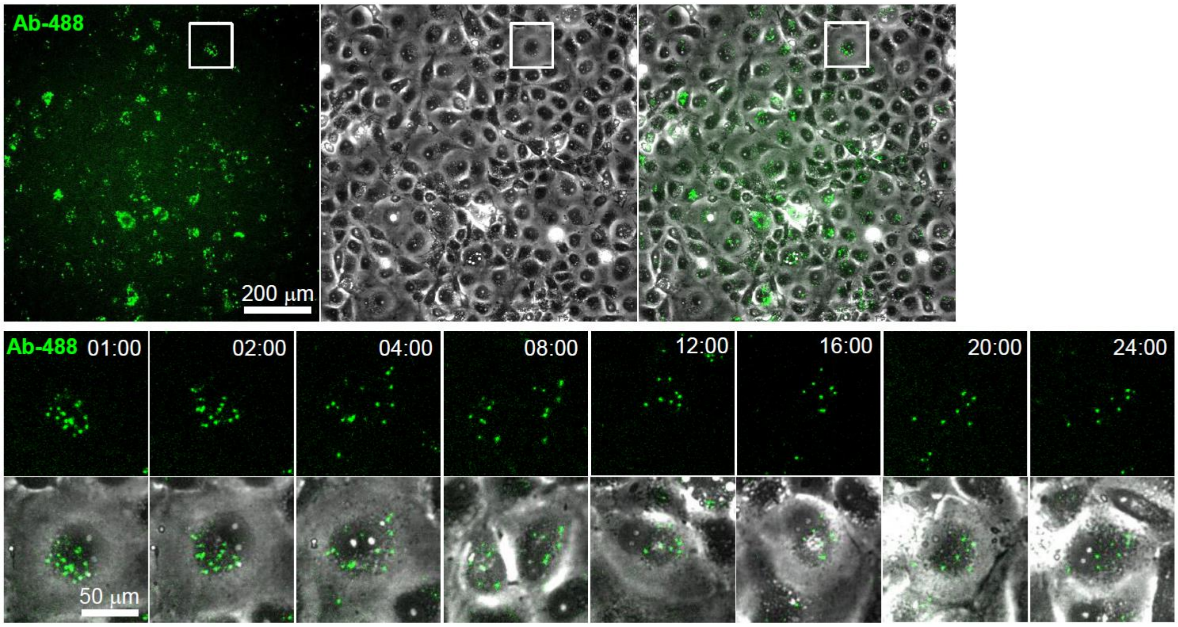

Ab-488
200 μm
Ab-488
01:00
02:00
04:00
08:00
12:00
16:00
20:00
24:00
50 μm

F

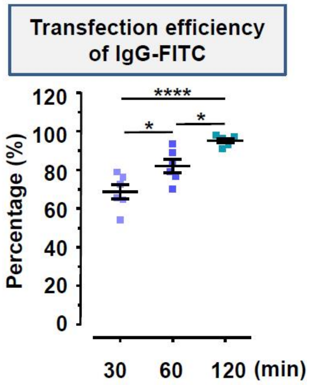

Transfection efficiency of IgG-FITC
Percentage (%)
120
100
80
60
40
20
0
****
*
*
30
60
120
(min)

G

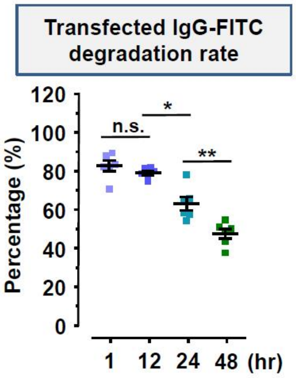

Transfected IgG-FITC degradation rate
Percentage (%)
120
100
80
60
40
20
0
n.s.
*
**
1
12
24
48
(hr)

H

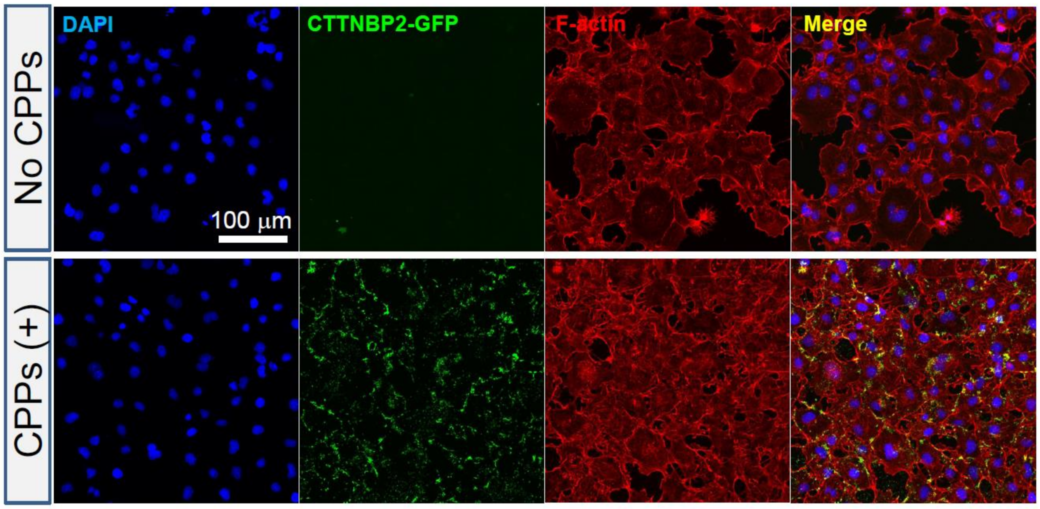

DAPI
CTTNBP2-GFP
F-actin
Merge
No CPPs
CPPs (+)
100 μm

Supplementary Figure S8. Teleofection enables the delivery of functional proteins into primary neurons.

(**A**) The flowchart illustrates the experimental design for generating distinct nanoparticles with various proteins intended for delivery into the same cells. (**B**) The representative images illustrate the nanoparticles included different anti-mouse antibodies (Ab) with unique fluorescent-dye conjugation, showing green for Ab-488, red for Ab-594, and magenta for Ab-647. The high magnification images show the details of each nanoparticle. (**C**) The flowchart indicates the experiment design to generate a single nanoparticle including different proteins. (**D**) The example images display that a single nanoparticle includes multiple anti-mouse antibodies, showing three different fluorescent signals. The high magnification images illustrate the details of each nanoparticle. The protein-CaP-nanoparticles generated by teleofection display a size in nanoscale. (**E**) The representative images depict the quantity of anti-mouse antibodies (Ab) labeled with green fluorescent dyes (Ab-488, shown in green) in COS7 cells following protein transfection via teleofection. The high magnification images reveal the alterations in Ab-488 within a COS7 cell over a time-dependent course. The bright-field images provide detailed views of cell morphology. (**F**-**G**) The dot plot graphs represent the quantitative data of transfection efficiency and degradation rate of Ab-488 in COS7 cells. (**H**) The example images indicate that CTTNBP2-GFP (green) enables to be delivered into COS7 cells as the existence of calcium. The nuclei and actin filament was outlined by DAPI and phalloidin in blue and red, respectively. The data were analyzed from six different areas of three independent experiments. Statistic: One-way ANOVA, (**F** and **G**). Values represent the mean ± s.e.m., *$P < 0.05$, **$P < 0.01$, ***$P < 0.001$, ****$P < 0.0001$. (These data are correlated to Figure 1 and 5).

Supplementary Figure S9

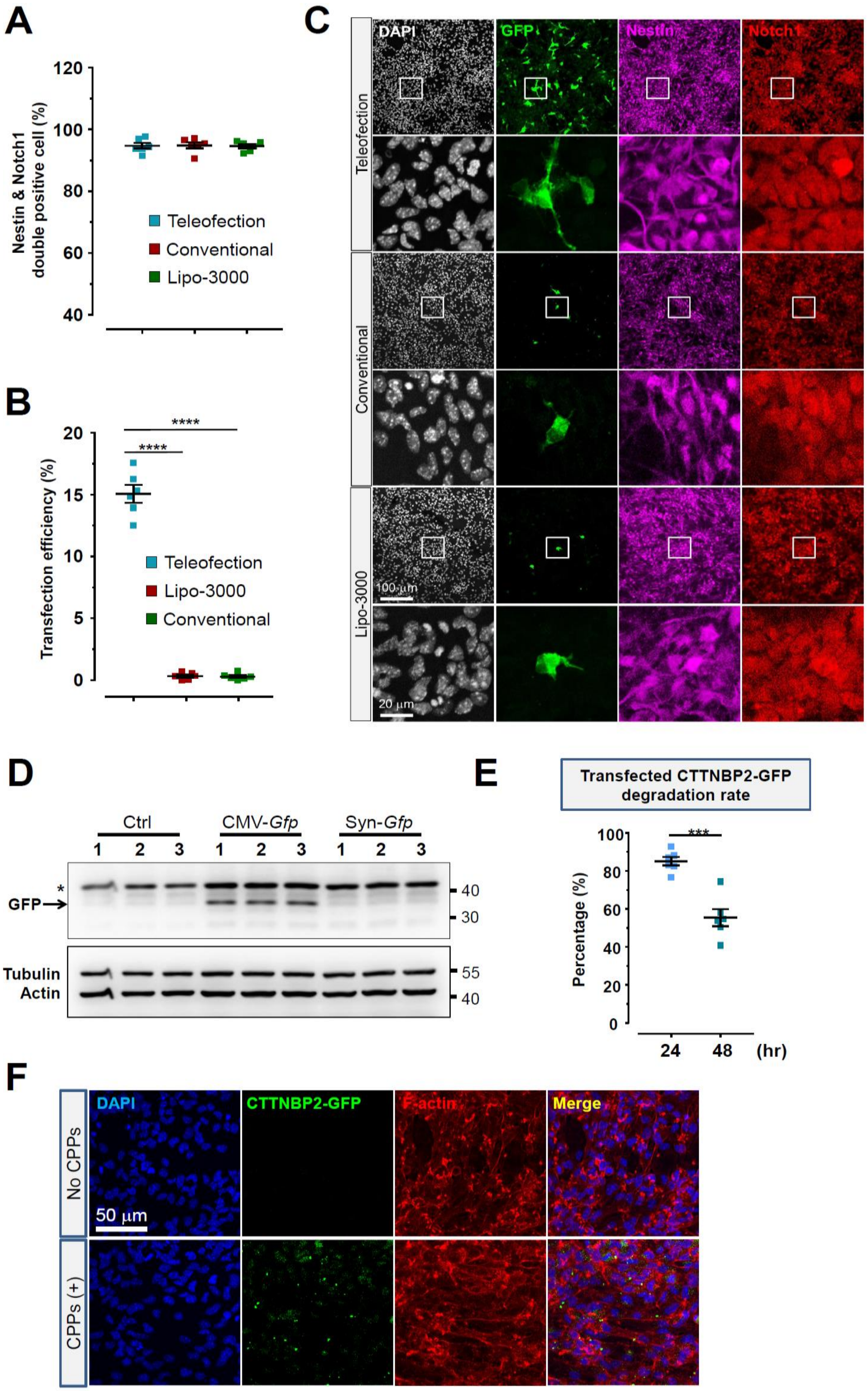

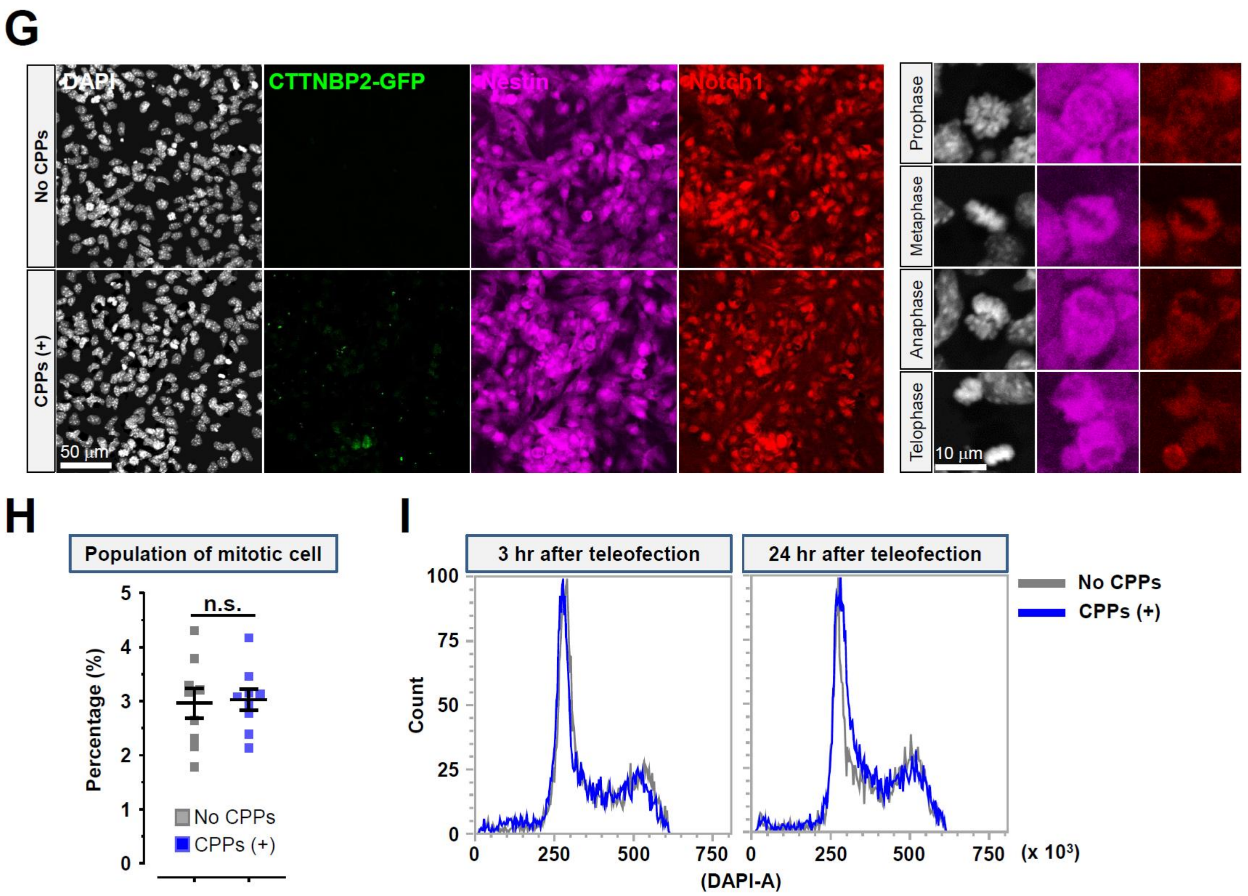

Supplementary Figure S9. Characterizing the capabilities and impacts of teleofection in NSCs.

(**A**-**C**) The dot plot graphs indicate the population of Nestin and Notch1 double-positive cells and the transfection efficiency of indicated protocols in NSCs. The example images show the numbers of GFP-positive NSCs under three different transfection protocols. (**D**) The data of Western blotting displays the expression level of GFP controlled by different promoter systems. (**E**) The dot plot graph represents the time-dependent change of CTTNBP2-GFP in NSCs after teleofection. (**F**) The examples of images indicate that CTTNBP2-GFP (green) can be delivered into NSCs as the existence of calcium. The nuclei and actin filament was outlined by DAPI and phalloidin in blue and red, respectively. (**G**) The representative images illustrate the effect of CaP-nanoparticles on the dividing of NSCs, 3 h after teleofection. The high magnification images show the various chromosome morphology at different mitosis state of NSCs. (**H**) The quantitative data of mitotic index from **G** indicates no significant effect of CaP-nanoparticles on the

dividing of NSCs. Nine regions selected from three independent coverslips was analyzed for each group. (**I**) The line graphs from flow cytometry show the effect of CaP-nanoparticles on the dividing of 293T cells. Statistic: One-way ANOVA, (**A** and **B**); Student's unpaired t-test (**E** and **H**). Values represent the mean ± s.e.m., **P* < 0.05, ***P* < 0.01, ****P* < 0.001, *****P* < 0.0001. (These data are correlated to Figure 6).

Table S1. Comparison of teleofection to the most common used DNA transfection methods. [1-9] [10-12] [13-15] [16-18] [19,20] [21,22] [23,24] [25] [26,27] [28,29] [30,31] [32,33]

	Methodological classification	Delivered cargo	Efficiency	Expense	Time and Labor	Biosafety Level	Customizable dosage and duration	Advantages	Limitations	Note	Supplementary References
Chemical methods	**Teleofection**	DNA<br>RNA<br>Protein	High	Very Low	Much less	1	V	Extremely low cytotoxicity<br>Higher biocompatible<br>Available for serial or multiple transfection<br>Suitble for primary neurons & neural stem cells<br>Rare aspine neuron & glial transfection<br>Available for biochemistry & molecular bioogy assays<br>Available for long-term incubation & living cell recording		Possibility for ex vivo & in vivo transfection	This article
	**CaP (conventional)**	DNA	Low	Very Low	Less	1	V	Low cytotoxicity (user dependent)	Unavailable for neural stem cell delivery<br>Nucleic acid delivery only; unavailable for protein delivery		1-9
	**Cationic lipid (Lipofection)**	DNA<br>RNA<br>Protein	Low	Low	Less	1	V		Potential cytotoxicity<br>Unavailable for neural stem cell delivery		10-12
	**Cationic polymer**	DNA<br>RNA<br>Protein	Low	Various	Less	1	V	Biocompatible	Potential cytotoxicity<br>Unavailable for neural stem cell delivery		13-15
Mechanical methods	**Microinjection**	DNA<br>RNA<br>Protein	High	High	More	1		Individual cell transfection<br>Precise dose	Plasma membrane stress	Possibility for nuclear injection	16-18
	**Biological ballistics (Biolistics or gene gun)**	DNA<br>RNA	Low	High	Moderate	1		Individual cell transfection	Cell damage<br>Restricted by tissue depth		19-20
	**Magnetic nanobeads**	DNA<br>RNA	Moderate	Low	Less	1	V		Poor dispersion		21-22
	**Electroporation**	DNA<br>RNA	Moderate	High	More	1			Cell damage<br>Skillful technician needed	Possibility for nuclear injection	23-24
	**Optical transfection (Laser-mediated poration)**	DNA<br>RNA	Low	High	More	1		High spatial resolution<br>Minimal cell damage	Restricted by tissue depth and light scattering	Possibility for both individual and large-scale transfection	25
Biological methods (viral transduction)	**Adenoviruses**	DNA	Very high	Moderate	Less	2			Possible immune responses	Each stereotype of virus has a unique affinity for specific subset of neuron populations	26-27
	**Adeno-associated viruses (AAVs)**	DNA	Very high	Moderate	Less	2		Fewer immune responses than adenovirus	Low cloning capacity<br>Possible humoral immune response		28-29
	**Lentiviruses**	RNA	Very high	Moderate	Less	2		Long duration of gene expression	Possible retroviral genotoxicity	Other types of retroviruses are unvalibale for non-dividing cells	30-31
	**Herpes simplex viruses (HSVs)**	DNA	Very high	Moderate	Few	2		High cloning capacity	Potential cytotoxicity<br>Possible immune responses	Retrograde axonal transport to trace neural connections	32-33

Table S2. Summary of main synthesis methods for CaP-nanoparticles.

8,34,35 36-38 39,40 41-43 44,45 46,47

Item	Method	Size	Morphology	Advantages	Limitations	Supplementary References
**1**	Teleofection	Nano	Particle	Low cost, large-scale, without special regents and equipments		This article
**2**	Sol-gel chemistry	Nano to micron	Particle/ sheet	Narrow size distribution, comparatively low synthesis temperature	High cost, serious aggregation, usually needs special regents, upscaling can be difficult	8, 34-35
**3**	Flame spray pyrolysis	Submicron to hundreds of microns	Particle	Rapid synthesis, large-scale, usually resulted in spheic structure	Difficult to obtain nano-size product, high energy consumption, special equipment necessary, no incorporation of organic molecules possible	36-38
**4**	Solid-state reactions	Micron	Diverse	Low cost, large-scale, without special regents and equipments	Severe aggregation, poor redispersability, application of organic compounds possible only after the synthesis	39-40
**5**	Wet-chemical precipitation (room temperature)	Nano	Diverse	Low cost, incorporation of compounds, often only water as solvent	Upscaling can be difficult and requires a continuous process	41-43
**6**	Wet-chemical precipitation (heat conditions)	Nano	Particle	High reaction rate and efficiency	Energy consumption; cannot load drugs (nucleic acids or proteins)	44-45
**7**	Pulsed laser ablation	Nano	Diverse	Control over product properties possible by adjustable laser parameters	Tendency for particle agglomeration; high-end laser equipment needed; difficult scale-up	46-47

Table S3. Summary of conventional methods for CaP-particles generation.

1 2 3 4 5 6 7 8

Item	Initial [Ca^{2+}]	Mixing method	Incubation time of nanoparticles	Transfection duration	Wash time	Note	Supplementary References
**1**	250 mM	1000-rpm vortexing	No data	No data	No data	For cell lines; CaP includes citrate anion and cationic poly-L-lysine	1
**2**	250 mM	No data	No data	2-6 hr	No data	For cell lines	2
**3**	2500 mM	Mild vortexing	1 min	6-7 hr	No data	For cell lines	3
**4**	248 mM	Dropwise with 600-rpm vortexing	15–20 min	45 min - 3 hr	15-20 min	For low-density neuronal cultures	4
**5**	248 mM	No data	30 min	7 hr	3 min	High amount DNA is required; pre-incubation with polyornithineis need; no research data	5
**6**	250 mM	Pipetting 10 times	30 sec	20–30 min	10 min	1 hr transfection causes cytotoxicity	6
**7**	250 mM	Pipetting once	No data	90 min	5-10 min	Enables biochemical approaches	7
**8**	250 mM	Dropwise adding	30 min	1 hr	3 times	For electrophysiological experiments	8
**9**	250 mM	No data	31 min	2 hr	2 times	For low-density neuronal cultures	9

Table S4. The volumes and the most common sizes for the transfection experiment.

Culture Plate	Culture area (cm^2)/ well	Multiplication time	Cell density (cells)/ well	Transfection mixture (μl)/ well	NB medium for transfection (μl)/ well	Culture medium (μl)/ well
96-well	0.32	0.0825	3x10^4	17.5	35	100~150
48-well	0.95	0.25	1x10^5	50	100	250~400
24-well	1.9	0.5	2x10^5	100	200	500~750
12-well	3.8	1	4x10^5	200	400	1000~1500
6-well	9.5	2.5	1x10^6	500	1000	2000~3000
60-mm	21	5.525	2.2x10^6	1105	2210	5000~7500
10-cm	55	14.475	5.8x10^6	2895	5790	10000~15000

Table S5. The plasmids information.

48-50

Item	Plasmid	Application	Figures	Supplementary References
1	pLL3.7-Syn-Gfp	Neuron & NSCs transfection	Fig. 2-5 and S2-7	48
2	pLL3.7-syn-Gfp-Cmtr1	Neuronal transfection	Fig. S3	48
3	pLL3.7-syn-mCherry	Neuronal transfection	Fig. 4, 5, S2, and S3	48
4	pLL3.7-Syn-siCmtr1-mCherry	Gene knockdown	Fig. 4	48
5	pLL3.7-Syn-siCPEB2	Gene knockdown	Fig. S7	49
6	pGL3	Luminescence reporter assay	Fig. 4 and 4	50
7	pGL3-PDGFRα-3'-UTR	Luminescence reporter assay	Fig. 4 and 6	50
8	pLL3.7-CMV-Gfp	NSC transfection	Fig. 6 and S9	48
9	pGW-HA-Sbf1	Neuronal transfection	Fig. S3	This study

Table S6. Current methods for RNA transfection in neurons and NSCs.

51-56

Item	Methods	Experimental Condition	Cell Types	Efficiency	Reporter Peak (hr)	Advantage	Disdvantage	Note	Ref.
1	Teleofection	*In vitro*	Primary neurons	Positive	8~16	Detectable by immunoblotting	Lower expression intensity		Fig. 4D
2	Lipofection (Lipo-2000)	*In vivo*	NSCs	<5%	n.d.	n.d.	Cytotoxicity in high conc.	RNA modification & CQ addition enhances transfection	51
3	Lipofection (TransIT)			15-35%	24	n.d.			
4	Lipofection (Lipo-2000)	*In vitro*	Primary neurons	25%	6		Cytotoxicity in high conc.		52
5	Lipofection (SNPs)	*In vivo*	$NeuN^+$ neurons	50%	24	High transfection distance	Targeting to both neurons & glia	Intracerebral injection to bypass BBB	53
6	Electroporation	*In vivo*	NSCs<br>Cortical neurons	60%	6	More efficient than DNA electroporation	Cause mechanical cell death	Preferential targeting to neurons	54
7	Lipofection (SNPs)	*In vivo*	$MAP2^+$ neurons	Positive	n.d.	Improved biocompatibility	n.d.	Preferential targeting to neurons	55
8	Lipofection (SNPs)	*In vivo*	Various neurons	Positive	n.d.	Long-lasting RNA distribution		Intranasal injection to bypass BBB	56
9	Cationic polymer (PEG)	*In vitro*	Primary neurons	Positive	n.d.	Improved biocompatibility	n.d.	n.d.	57
10		*In vivo*	$NeuN^+$ neurons	Positive	4		n.d.	n.d.	

Supplementary References

1 Khan, M. A., Wu, V. M., Ghosh, S. & Uskokovic, V. Gene delivery using calcium phosphate nanoparticles: Optimization of the transfection process and the effects of citrate and poly(l-lysine) as additives. *Journal of Colloid & Interface Science* **471**, 48-58 (2016).

2 Jordan, M., Schallhorn, A. & Wurm, F. M. Transfecting mammalian cells: Optimization of critical parameters affecting calcium-phosphate precipitate formation. *Nucleic Acids Research* **24**, 596-601, doi:Doi 10.1093/Nar/24.4.596 (1996).

3 Guo, L. *et al.* Optimizing conditions for calcium phosphate mediated transient transfection. *Saudi J Biol Sci* **24**, 622-629, doi:10.1016/j.sjbs.2017.01.034S1319-562X(17)30043-8 [pii] (2017).

4 Jiang, M. & Chen, G. High Ca2+-phosphate transfection efficiency in low-density neuronal cultures. *Nat Protoc* **1**, 695-700, doi:nprot.2006.86 [pii]10.1038/nprot.2006.86 (2006).

5 Watson, A. & Latchman, D. Gene Delivery into Neuronal Cells by Calcium Phosphate-Mediated Transfection. *Methods* **10**, 289-291, doi:doi.org/10.1006/meth.1996.0105 (1996).

6 Wang, S. & Cho, Y. K. An Optimized Calcium-Phosphate Transfection Method for Characterizing Genetically Encoded Tools in Primary Neurons. *Methods Mol Biol* **1408**, 243-249, doi:10.1007/978-1-4939-3512-3_16 (2016).

7 Goetze, B., Grunewald, B., Baldassa, S. & Kiebler, M. Chemically controlled formation of a DNA/calcium phosphate coprecipitate: application for transfection of mature hippocampal neurons. *J Neurobiol* **60**, 517-525, doi:10.1002/neu.20073 (2004).

8 Watanabe, S. Y. *et al.* Calcium phosphate-mediated transfection of primary cultured brain neurons using GFP expression as a marker: application for single neuron electrophysiology. *Neurosci Res* **33**, 71-78, doi:S0168-0102(98)00113-8 [pii]10.1016/s0168-0102(98)00113-8 (1999).

9 Kohrmann, M. *et al.* Fast, convenient, and effective method to transiently transfect primary hippocampal neurons. *J Neurosci Res* **58**, 831-835 (1999).

10 Holt, C. E., Garlick, N. & Cornel, E. Lipofection of Cdnas in the Embryonic Vertebrate Central-Nervous-System. *Neuron* **4**, 203-214, doi:Doi 10.1016/0896-6273(90)90095-W (1990).

11 Ohki, E. C., Tilkins, M. L., Ciccarone, V. C. & Price, P. J. Improving the transfection efficiency of post-mitotic neurons. *J Neurosci Methods* **112**, 95-99, doi:S0165027001004411 [pii]10.1016/s0165-0270(01)00441-1 (2001).

12 Wang, T., Larcher, L. M., Ma, L. X. & Veedu, R. N. Systematic Screening of Commonly Used Commercial Transfection Reagents towards Efficient Transfection of Single-Stranded Oligonucleotides. *Molecules* **23**, doi:Artn 256410.3390/Molecules23102564 (2018).

13 Boussif, O. *et al.* A Versatile Vector for Gene and Oligonucleotide Transfer into Cells in Culture and in-Vivo - Polyethylenimine. *P Natl Acad Sci USA* **92**, 7297-7301, doi:DOI 10.1073/pnas.92.16.7297 (1995).

14 Abdallah, B. *et al.* A powerful nonviral vector for in vivo gene transfer into the adult mammalian brain: Polyethylenimine. *Hum Gene Ther* **7**, 1947-1954, doi:DOI 10.1089/hum.1996.7.16-1947 (1996).

15 Tonges, L. *et al.* Stearylated octaarginine and artificial virus-like particles for transfection of siRNA into primary rat neurons. *Rna* **12**, 1431-1438, doi:10.1261/rna.2252206 (2006).

16 Zhang, Y., Goodyer, C. & LeBlanc, A. Selective and protracted apoptosis in human primary neurons microinjected with active caspase-3,-6,-7, and-8. *Journal of Neuroscience* **20**, 8384-8389, doi:Doi 10.1523/Jneurosci.20-22-08384.2000 (2000).

17 Zhang, Y. *et al.* p75 neurotrophin receptor protects primary cultures of human neurons against extracellular amyloid beta peptide cytotoxicity. *Journal of Neuroscience* **23**, 7385-7394 (2003).

18 Schratt, G. M. *et al.* A brain-specific microRNA regulates dendritic spine development. *Nature* **439**, 283-289, doi:10.1038/nature04367 (2006).

19 Lo, D. C., Mcallister, A. K. & Katz, L. C. Neuronal Transfection in Brain-Slices Using Particle-Mediated Gene-Transfer. *Neuron* **13**, 1263-1268, doi:Doi 10.1016/0896-6273(94)90412-X (1994).
20 Dib-Hajj, S. D. *et al.* Transfection of rat or mouse neurons by biolistics or electroporation. *Nat Protoc* **4**, 1118-1127, doi:10.1038/nprot.2009.90 (2009).
21 Buerli, T. *et al.* Efficient transfection of DNA or shRNA vectors into neurons using magnetofection. *Nat Protoc* **2**, 3090-3101, doi:10.1038/nprot.2007.445 (2007).
22 Khoo, K. S. *et al.* Nanomaterials Utilization in Biomass for Biofuel and Bioenergy Production. *Energies* **13**, doi:Artn 89210.3390/En13040892 (2020).
23 Zeitelhofer, M. *et al.* High-efficiency transfection of mammalian neurons via nucleofection. *Nat Protoc* **2**, 1692-1704, doi:10.1038/nprot.2007.226 (2007).
24 Zeitelhofer, M. *et al.* High-efficiency transfection of mammalian neurons via nucleofection. *Nat Protoc* **2**, 1692-1704, doi:nprot.2007.226 [pii]10.1038/nprot.2007.226 (2007).
25 Barrett, L. E. *et al.* Region-directed phototransfection reveals the functional significance of a dendritically synthesized transcription factor. *Nat Methods* **3**, 455-460, doi:10.1038/NMETH885 (2006).
26 Lasalle, G. L. *et al.* An Adenovirus Vector for Gene-Transfer into Neurons and Glia in the Brain. *Science* **259**, 988-990, doi:DOI 10.1126/science.8382374 (1993).
27 Harding, T. C., Geddes, B. J., Murphy, D., Knight, D. & Uney, J. B. Switching transgene expression in the brain using an adenoviral tetracycline-regulatable system. *Nat Biotechnol* **16**, 553-555, doi:Doi 10.1038/Nbt0698-553 (1998).
28 Royo, N. C. *et al.* Specific AAV serotypes stably transduce primary hippocampal and cortical cultures with high efficiency and low toxicity. *Brain Res* **1190**, 15-22, doi:10.1016/j.brainres.2007.11.015 (2008).
29 Chakrabarty, P. *et al.* Capsid Serotype and Timing of Injection Determines AAV Transduction in the Neonatal Mice Brain. *Plos One* **8**, doi:ARTN e6768010.1371/journal.pone.0067680 (2013).
30 Blomer, U. *et al.* Highly efficient and sustained gene transfer in adult neurons with a lentivirus vector. *J Virol* **71**, 6641-6649 (1997).
31 Naldini, L., Blomer, U., Gage, F. H., Trono, D. & Verma, I. M. Efficient transfer, integration, and sustained long-term expression of the transgene in adult rat brains injected with a lentiviral vector. *P Natl Acad Sci USA* **93**, 11382-11388, doi:DOI 10.1073/pnas.93.21.11382 (1996).
32 Zemanick, M. C., Strick, P. L. & Dix, R. D. Direction of Transneuronal Transport of Herpes-Simplex Virus-1 in the Primate Motor System Is Strain-Dependent. *P Natl Acad Sci USA* **88**, 8048-8051, doi:DOI 10.1073/pnas.88.18.8048 (1991).
33 Geller, A. I. & Freese, A. Infection of Cultured Central-Nervous-System Neurons with a Defective Herpes-Simplex Virus-1 Vector Results in Stable Expression of Escherichia-Coli Beta-Galactosidase. *P Natl Acad Sci USA* **87**, 1149-1153, doi:DOI 10.1073/pnas.87.3.1149 (1990).
34 Ishikawa, K., Garskaite, E. & Kareiva, A. Sol-gel synthesis of calcium phosphate-based biomaterials-A review of environmentally benign, simple, and effective synthesis routes. *J Sol-Gel Sci Techn* **94**, 551-572, doi:10.1007/s10971-020-05245-8 (2020).
35 de Oliveira, L. K., Molina, E. F., Moura, A. L. A., de Faria, E. H. & Ciuffi, K. J. Synthesis, Characterization, and Environmental Applications of Hybrid Materials Based on Humic Acid Obtained by the Sol Gel Route. *Acs Appl Mater Inter* **8**, 1478-1485, doi:10.1021/acsami.5b10810 (2016).
36 Caballero, S. S. R. *et al.* Combination of biocompatible hydrogel precursors to apatitic calcium phosphate cements (CPCs): Influence of the in situ hydrogel reticulation on theCPCproperties. *J Biomed Mater Res B* **109**, 102-116, doi:10.1002/jbm.b.34685 (2021).

37 Goldberg, M. A. *et al.* In situ magnesium calcium phosphate cements formation: From one pot powders precursors synthesis to in vitro investigations. *Bioact Mater* **5**, 644-658, doi:10.1016/j.bioactmat.2020.03.011 (2020).
38 Cho, J. S., Ko, Y. N., Koo, H. Y. & Kang, Y. C. Synthesis of nano-sized biphasic calcium phosphate ceramics with spherical shape by flame spray pyrolysis. *J Mater Sci-Mater M* **21**, 1143-1149, doi:10.1007/s10856-009-3980-1 (2010).
39 Cleries, L. *et al.* Mechanical properties of calcium phosphate coatings deposited by laser ablation. *Biomaterials* **21**, 967-971, doi:Doi 10.1016/S0142-9612(99)00240-9 (2000).
40 Guo, X. J. *et al.* Effect of calcining temperature on particle size of hydroxyapatite synthesized by solid-state reaction at room temperature. *Adv Powder Technol* **24**, 1034-1038, doi:10.1016/j.apt.2013.03.002 (2013).
41 Kusnieruk, S. *et al.* Influence of hydrothermal synthesis parameters on the properties of hydroxyapatite nanoparticles. *Beilstein J Nanotech* **7**, 1586-1601, doi:10.3762/bjnano.7.153 (2016).
42 Jacobs, E. E., Gronowicz, G., Hurley, M. M. & Kuhn, L. T. Biomimetic calcium phosphate/polyelectrolyte multilayer coatings for sequential delivery of multiple biological factors. *J Biomed Mater Res A* **105**, 1500-1509, doi:10.1002/jbm.a.35985 (2017).
43 Welzel, T., Meyer-Zaika, W. & Epple, M. Continuous preparation of functionalised calcium phosphate nanoparticles with adjustable crystallinity. *Chem Commun*, 1204-1205, doi:10.1039/b402521k (2004).
44 Qi, C. *et al.* Hydroxyapatite Hierarchically Nanostructured Porous Hollow Microspheres: Rapid, Sustainable Microwave-Hydrothermal Synthesis by Using Creatine Phosphate as an Organic Phosphorus Source and Application in Drug Delivery and Protein Adsorption. *Chem-Eur J* **19**, 5332-5341, doi:10.1002/chem.201203886 (2013).
45 Yu, W. L. *et al.* Copper-doped mesoporous hydroxyapatite microspheres synthesized by a microwave-hydrothermal method using creatine phosphate as an organic phosphorus source: application in drug delivery and enhanced bone regeneration. *Journal of Materials Chemistry B* **5**, 1039-1052, doi:10.1039/c6tb02747d (2017).
46 Tveryanovich, Y. S., Manshina, A. A. & Tverjanovich, A. S. Production of nanodispersed materials and thin films by laser ablation techniques in liquid and in vacuum. *Russ Chem Rev+* **81**, 1091-1116, doi:10.1070/RC2012v081n12ABEH004285 (2012).
47 Boutinguiza, M., Comesana, R., Lusquinos, F., Riveiro, A. & Pou, J. Production of nanoparticles from natural hydroxylapatite by laser ablation. *Nanoscale Res Lett* **6**, doi:Artn 25510.1186/1556-276x-6-255 (2011).
48 Lee, Y. L., Kung, F. C., Lin, C. H. & Huang, Y. S. CMTR1-Catalyzed 2'-O-Ribose Methylation Controls Neuronal Development by Regulating Camk2alpha Expression Independent of RIG-I Signaling. *Cell Rep* **33**, 108269, doi:S2211-1247(20)31258-4 [pii]10.1016/j.celrep.2020.108269 (2020).
49 Lu, W. H., Yeh, N. H. & Huang, Y. S. CPEB2 Activates GRASP1 mRNA Translation and Promotes AMPA Receptor Surface Expression, Long-Term Potentiation, and Memory. *Cell Rep* **21**, 1783-1794, doi:S2211-1247(17)31536-X [pii]10.1016/j.celrep.2017.10.073 (2017).
50 Lai, Y. T. *et al.* CPEB2-activated PDGFRalpha mRNA translation contributes to myofibroblast proliferation and pulmonary alveologenesis. *J Biomed Sci* **27**, 52, doi:10.1186/s12929-020-00643-010.1186/s12929-020-00643-0 [pii] (2020).
51 Khayamabed, R. *et al.* Efficient Modified-mRNA Transfection in Neural Stem Cells. *Physiol Pharmacol* **27**, 80-91, doi:10.52547/phypha.27.1.5 (2023).

52 Williams, D. J., Puhl, H. L. & Ikeda, S. R. A simple, highly efficient method for heterologous expression in mammalian primary neurons using cationic lipid-mediated mRNA transfection. *Front Neurosci-Switz* **4**, doi:Artn 18110.3389/Fnins.2010.00181 (2010).
53 Tuma, J. *et al.* Lipid Nanoparticles Deliver mRNA to the Brain after an Intracerebral Injection. *Biochemistry*, doi:10.1021/acs.biochem.3c00371 (2023).
54 Bugeon, S. *et al.* Direct and efficient transfection of mouse neural stem cells and mature neurons by in vivo mRNA electroporation. *Development* **144**, 3968-3977, doi:dev.151381 [pii]10.1242/dev.151381 (2017).
55 Qiu, M., Li, Y., Bloomer, H. & Xu, Q. Developing Biodegradable Lipid Nanoparticles for Intracellular mRNA Delivery and Genome Editing. *Acc Chem Res* **54**, 4001-4011, doi:10.1021/acs.accounts.1c00500 (2021).
56 Dhaliwal, H. K., Fan, Y., Kim, J. & Amiji, M. M. Intranasal Delivery and Transfection of mRNA Therapeutics in the Brain Using Cationic Liposomes. *Mol Pharm* **17**, 1996-2005, doi:10.1021/acs.molpharmaceut.0c00170 (2020).
